# Supplementary material for: Variation in excess all-cause mortality by age, sex, and province during the first wave of the COVID-19 pandemic in Italy
Source: Sci Rep. 2022 Jan 20;12:1077. doi: 10.1038/s41598-022-04993-7 (PMC8776797; doi:10.1038/s41598-022-04993-7)
Supplement: Supplementary file 1 — Supplementary Information. [file 41598_2022_4993_MOESM1_ESM.pdf]

*Appendix to: Variation in excess all-cause mortality by age, sex, and province during the first wave of the COVID-19 pandemic in Italy*

# Supplementary appendix

## Covariate preparation

Supplementary Table 1 lists the predictive covariates used to estimate baseline mortality over the study time period, 2015-2020.

**Supplementary Table 1**: Summary of predictive covariates fitted in the model for baseline mortality

| Covariate | Source | Resolution | Years covered |
|---|---|---|---|
| Total fertility rate | Istat[1] | Province, year | 2015-2018 |
| First quarter unemployment, age 15 and above | Istat[1] | Province, year, sex | 2015-2019 |
| Proportion of eligible households receiving at-home social services | Istat[1] | Province, year | 2015-2017 |
| Proportion of households with taxable annual income below 10,000 Euros | Istat[1] | Commune, year | 2015-2018 |
| Average driving time to the nearest health facility | Malaria Atlas Project[3] | 1 km x 1km (gridded) | Synoptic |
| Average elevation of residence | US Geological Survey[2] | 15 arc-second (gridded) | Synoptic |
| Mean daily temperature | Meteostat[4,5] | Point location, day | 2015-2020 |

Synoptic and single-year covariates were used across all years, while time-varying covariates not available for the final years of the time series were projected forwards by replicating values from the final observed year of data. Covariates observed at a more detailed spatial resolution than provinces were aggregated to the province level using a population-weighted mean across units within a province.

Supplementary Table 2 lists justifications for each of the seven predictive covariates included in the analysis.

**Supplementary Table 2**: Justification for covariate inclusion

| Covariate | Justification |
|---|---|
| Total fertility rate | The Global Burden of Disease cross-national systematic analysis identifies the total fertility rate as one of three highly-predictive covariates for all-cause mortality,[6] along with educational attainment and income. |
| First quarter unemployment, age 15 and above | A meta-analysis of 42 studies from primarily high-income countries found that unemployment was associated with increased risk of death among individuals aged 65 and below.[7] |
| Proportion of eligible households receiving at-home social services | Proxy for the uptake of social welfare programs across provinces and years. An analysis of OECD data found that increased social welfare spending per capita was associated with reductions in age-standardized all-cause mortality, after adjusting for country-specific factors,[8] although the association between social welfare spending and health outcomes may vary based on the analytical framework used.[9] |
| Proportion of households with taxable annual income below 10,000 Euros | Proxy for variation in household income across provinces. A Danish cohort study found that household income was associated with all-cause mortality rates after adjusting for individual risk factors.[10] The Global Burden of Disease Study identifies income as one of three highly-predictive covariates for all-cause mortality.[6] |
| Average driving time to the nearest health facility | An observational study in England found that greater distance to hospitals was associated with increased mortality for patients with medical emergencies.[11] |
| Average elevation of residence | Population-based ecological studies from the United States, Greece, and Switzerland, among other countries, suggest that increased altitude may be associated with lower mortality from cardiovascular disease and stroke, but higher mortality from COPD and lower respiratory infection.[12] |
| Mean daily temperature | A meta-analysis of 15 studies, primarily in high-income settings, identified a significant association between extreme temperature intervals and all-cause mortality.[13] |

### Weekly temperature covariate

Point estimates of daily temperature were downloaded for the three most populous pixels in each province using the Meteostat API. Because temperature was not recorded for all time periods and locations, the following process was used to fill temperatures across all provinces and weeks in the time series:

1. Find average weekly temperatures across all observed days by year, week, and observed location.
2. Interpolate by week. In cases where 3 consecutive weeks or fewer are missing between observed temperature values in a location, estimate missing temperatures by interpolating between the nearest observed weeks
3. Aggregate by province. For each province, year, and week of the year, average available observation across the three sampled locations.
4. In rare instances where all observations for a province were missing for a given province, year, and week, temperature was filled with recorded observations from neighboring provinces with a similar elevation and level of solar exposure.

Additional covariate preparation information and analytical code can be accessed online at https://github.com/njhenry/covidemr.

## Model validation

See the Methods section for an overview of the model validation process. We compared predictive validity metrics across six model specifications, denoted A through G. Each model specification was fit under two sets of conditions. For in-sample testing, we fit each model type using the full baseline mortality dataset from January 2015 through February 2020, and then compared estimates of underlying mortality rates with the observed data. However, the resulting in-sample metrics for goodness of fit can mask model overfitting, allowing overly flexible models to follow spurious local trends in the data.

To generate more robust estimates of model predictive validity, we also conducted out-of-sample testing that mimicked the data generation process for all-cause mortality data under a hypothetical baseline where no mortality shock occurred in 2020. Five data holdouts were created, where each holdout was missing observations from March through December in one of the five baseline years 2015-2019. We then fit each model specification based on all remaining observations, and then generated predictive validity metrics by comparing mortality rate estimates and observed data from only the held-out weeks.

As described in the Methods section, when the number of observed outcomes is low, it may be advantageous to aggregate the results across multiple dimensions of interest before generating predictive validity estimates. For this analysis, all predictive validity metrics were generated for individual observations (disaggregated by province, age group, year, and week) as well as grouped mortality rates across each province-year.

Supplementary Tables 3 and 4 report in-sample predictive validity metrics for the six model specifications when compared by province-age-year-week and by province-year groupings, respectively. Supplementary Tables 5 and 6 report out-of-sample predictive validity metrics for the six model specifications when compared by province-age-year-week and by province-year groupings, respectively. For all comparisons, root mean squared error, squared error relative to a simple mean of all observations, and squared error relative to a simpler model that uses the mean observed mortality rate for each age group were generated. We also report empirical coverage of the 50%, 80%, 90%, 95%, and 99% uncertainty intervals of each model type. Because all models are run by sex, sex-specific results as well as averages across male and female models are reported.

**Supplementary Table 3**: In-sample predictive validity metrics comparing model estimates to individual data observations. RMSE: root mean squared error. RSE: relative squared error.

| Model Specification | Number of Fourier terms | Seasonality fit separately by: | Sex | RMSE | RSE | RSE compared to age-specific mean | 50% UI | 80% UI | 90% UI | 95% UI | 99% UI |
|---|---|---|---|---|---|---|---|---|---|---|---|
| A | 3 | Age, province | Male | 9.774E-04 | 0.1571 | 0.8612 | 0.6174 | 0.8629 | 0.9351 | 0.9686 | 0.9929 |
| A | 3 | Age, province | Female | 5.723E-04 | 0.0913 | 0.8667 | 0.6335 | 0.8683 | 0.9372 | 0.9692 | 0.9924 |
| A | 3 | Age, province | (Average) | 7.748E-04 | 0.1242 | 0.8639 | 0.6255 | 0.8656 | 0.9362 | 0.9689 | 0.9927 |
| B | 2 | Age, province | Male | 9.806E-04 | 0.1581 | 0.8667 | 0.6182 | 0.8642 | 0.9365 | 0.9696 | 0.9932 |
| B | 2 | Age, province | Female | 5.744E-04 | 0.0920 | 0.8731 | 0.6344 | 0.8699 | 0.9392 | 0.9701 | 0.9928 |
| B | 2 | Age, province | (Average) | 7.775E-04 | 0.1250 | 0.8699 | 0.6263 | 0.8671 | 0.9379 | 0.9699 | 0.9930 |
| C | 1 | Age, province | Male | 9.908E-04 | 0.1614 | 0.8849 | 0.6080 | 0.8546 | 0.9281 | 0.9639 | 0.9910 |
| C | 1 | Age, province | Female | 5.866E-04 | 0.0960 | 0.9107 | 0.6223 | 0.8584 | 0.9295 | 0.9635 | 0.9895 |
| C | 1 | Age, province | (Average) | 7.887E-04 | 0.1287 | 0.8978 | 0.6151 | 0.8565 | 0.9288 | 0.9637 | 0.9903 |
| D | 3 | Province | Male | 9.942E-04 | 0.1625 | 0.8910 | 0.6085 | 0.8545 | 0.9298 | 0.9652 | 0.9917 |
| D | 3 | Province | Female | 5.850E-04 | 0.0954 | 0.9056 | 0.6263 | 0.8639 | 0.9335 | 0.9668 | 0.9916 |
| D | 3 | Province | (Average) | 7.896E-04 | 0.1290 | 0.8983 | 0.6174 | 0.8592 | 0.9317 | 0.9660 | 0.9917 |
| E | 2 | Province | Male | 9.945E-04 | 0.1626 | 0.8915 | 0.6067 | 0.8531 | 0.9283 | 0.9640 | 0.9914 |
| E | 2 | Province | Female | 5.820E-04 | 0.0944 | 0.8964 | 0.6300 | 0.8683 | 0.9375 | 0.9694 | 0.9927 |
| E | 2 | Province | (Average) | 7.882E-04 | 0.1285 | 0.8939 | 0.6184 | 0.8607 | 0.9329 | 0.9667 | 0.9921 |
| F | 1 | Province | Male | 9.997E-04 | 0.1643 | 0.9010 | 0.6028 | 0.8498 | 0.9250 | 0.9617 | 0.9906 |
| F | 1 | Province | Female | 5.945E-04 | 0.0985 | 0.9353 | 0.6246 | 0.8624 | 0.9327 | 0.9662 | 0.9913 |
| F | 1 | Province | (Average) | 7.971E-04 | 0.1314 | 0.9181 | 0.6137 | 0.8561 | 0.9288 | 0.9639 | 0.9909 |
| G | (No Fourier term) | N/A | Male | 1.005E-03 | 0.1661 | 0.9105 | 0.5995 | 0.8479 | 0.9242 | 0.9610 | 0.9903 |
| G | (No Fourier term) | N/A | Female | 6.021E-04 | 0.1011 | 0.9596 | 0.6151 | 0.8526 | 0.9264 | 0.9616 | 0.9890 |
| G | (No Fourier term) | N/A | (Average) | 8.036E-04 | 0.1336 | 0.9350 | 0.6073 | 0.8502 | 0.9253 | 0.9613 | 0.9897 |

**Supplementary Table 4**: In-sample predictive validity metrics comparing model estimates to observed data by province-year grouping. RMSE: root mean squared error. RSE: relative squared error.

| Model Specification | Number of Fourier terms | Seasonality fit separately by: | Sex | RMSE | RSE | 50% UI | 80% UI | 90% UI | 95% UI | 99% UI |
|---|---|---|---|---|---|---|---|---|---|---|
| A | 3 | Age, province | Male | 2.299E-05 | 0.2855 | 0.6426 | 0.9071 | 0.9577 | 0.9788 | 0.9943 |
| A | 3 | Age, province | Female | 2.547E-05 | 0.2729 | 0.6242 | 0.8929 | 0.9482 | 0.9725 | 0.9897 |
| A | 3 | Age, province | (Average) | 2.423E-05 | 0.2792 | 0.6334 | 0.9000 | 0.9530 | 0.9757 | 0.9920 |
| B | 2 | Age, province | Male | 2.268E-05 | 0.2778 | 0.6730 | 0.9210 | 0.9654 | 0.9831 | 0.9948 |
| B | 2 | Age, province | Female | 2.525E-05 | 0.2683 | 0.6532 | 0.9072 | 0.9590 | 0.9768 | 0.9920 |
| B | 2 | Age, province | (Average) | 2.396E-05 | 0.2730 | 0.6631 | 0.9141 | 0.9622 | 0.9800 | 0.9934 |
| C | 1 | Age, province | Male | 2.453E-05 | 0.3250 | 0.6064 | 0.8722 | 0.9363 | 0.9618 | 0.9848 |
| C | 1 | Age, province | Female | 2.768E-05 | 0.3225 | 0.5764 | 0.8487 | 0.9222 | 0.9527 | 0.9791 |
| C | 1 | Age, province | (Average) | 2.611E-05 | 0.3237 | 0.5914 | 0.8604 | 0.9292 | 0.9572 | 0.9819 |
| D | 3 | Province | Male | 2.332E-05 | 0.2937 | 0.6245 | 0.8980 | 0.9548 | 0.9780 | 0.9943 |
| D | 3 | Province | Female | 2.659E-05 | 0.2974 | 0.6203 | 0.8888 | 0.9501 | 0.9735 | 0.9916 |
| D | 3 | Province | (Average) | 2.495E-05 | 0.2956 | 0.6224 | 0.8934 | 0.9524 | 0.9757 | 0.9930 |
| E | 2 | Province | Male | 2.364E-05 | 0.3019 | 0.6117 | 0.8888 | 0.9489 | 0.9743 | 0.9925 |
| E | 2 | Province | Female | 2.584E-05 | 0.2809 | 0.6534 | 0.9131 | 0.9642 | 0.9813 | 0.9958 |
| E | 2 | Province | (Average) | 2.474E-05 | 0.2914 | 0.6325 | 0.9010 | 0.9565 | 0.9778 | 0.9941 |
| F | 1 | Province | Male | 2.443E-05 | 0.3223 | 0.5934 | 0.8625 | 0.9308 | 0.9626 | 0.9865 |
| F | 1 | Province | Female | 2.765E-05 | 0.3218 | 0.6027 | 0.8805 | 0.9463 | 0.9714 | 0.9884 |
| F | 1 | Province | (Average) | 2.604E-05 | 0.3220 | 0.5981 | 0.8715 | 0.9386 | 0.9670 | 0.9874 |
| G | (No Fourier term) | N/A | Male | 2.491E-05 | 0.3351 | 0.5690 | 0.8431 | 0.9233 | 0.9589 | 0.9854 |
| G | (No Fourier term) | N/A | Female | 2.862E-05 | 0.3446 | 0.5340 | 0.8234 | 0.9137 | 0.9491 | 0.9808 |
| G | (No Fourier term) | N/A | (Average) | 2.676E-05 | 0.3398 | 0.5515 | 0.8332 | 0.9185 | 0.9540 | 0.9831 |

**Supplementary Table 5**: Out-of-sample predictive validity metrics comparing model estimates to individual data observations. RMSE: root mean squared error. RSE: relative squared error.

| Model Specification | Number of Fourier terms | Seasonality fit separately by: | Sex | RMSE | RSE | RSE compared to age-specific mean | 50% UI | 80% UI | 90% UI | 95% UI | 99% UI |
|---|---|---|---|---|---|---|---|---|---|---|---|
| A | 3 | Age, province | Male | 9.756E-04 | 0.1727 | 0.9154 | 0.6172 | 0.8643 | 0.9364 | 0.9688 | 0.9931 |
| A | 3 | Age, province | Female | 5.683E-04 | 0.1004 | 0.9322 | 0.6370 | 0.8733 | 0.9409 | 0.9713 | 0.9937 |
| A | 3 | Age, province | (Average) | 7.720E-04 | 0.1365 | 0.9238 | 0.6271 | 0.8688 | 0.9387 | 0.9700 | 0.9934 |
| B | 2 | Age, province | Male | 9.710E-04 | 0.1710 | 0.9067 | 0.6257 | 0.8711 | 0.9406 | 0.9715 | 0.9942 |
| B | 2 | Age, province | Female | 5.660E-04 | 0.0996 | 0.9245 | 0.6441 | 0.8791 | 0.9458 | 0.9741 | 0.9940 |
| B | 2 | Age, province | (Average) | 7.685E-04 | 0.1353 | 0.9156 | 0.6349 | 0.8751 | 0.9432 | 0.9728 | 0.9941 |
| C | 1 | Age, province | Male | 9.723E-04 | 0.1715 | 0.9092 | 0.6195 | 0.8657 | 0.9370 | 0.9694 | 0.9931 |
| C | 1 | Age, province | Female | 5.733E-04 | 0.1021 | 0.9484 | 0.6402 | 0.8752 | 0.9416 | 0.9716 | 0.9931 |
| C | 1 | Age, province | (Average) | 7.728E-04 | 0.1368 | 0.9288 | 0.6299 | 0.8705 | 0.9393 | 0.9705 | 0.9931 |
| D | 3 | Province | Male | 9.695E-04 | 0.1705 | 0.9040 | 0.6320 | 0.8736 | 0.9413 | 0.9723 | 0.9939 |
| D | 3 | Province | Female | 5.824E-04 | 0.1054 | 0.9787 | 0.6521 | 0.8848 | 0.9479 | 0.9755 | 0.9946 |
| D | 3 | Province | (Average) | 7.759E-04 | 0.1380 | 0.9414 | 0.6420 | 0.8792 | 0.9446 | 0.9739 | 0.9942 |
| E | 2 | Province | Male | 9.645E-04 | 0.1688 | 0.8946 | 0.6269 | 0.8708 | 0.9404 | 0.9709 | 0.9936 |
| E | 2 | Province | Female | 5.665E-04 | 0.0998 | 0.9262 | 0.6506 | 0.8830 | 0.9467 | 0.9742 | 0.9944 |
| E | 2 | Province | (Average) | 7.655E-04 | 0.1343 | 0.9104 | 0.6388 | 0.8769 | 0.9436 | 0.9725 | 0.9940 |
| F | 1 | Province | Male | 9.745E-04 | 0.1723 | 0.9134 | 0.6266 | 0.8700 | 0.9391 | 0.9705 | 0.9933 |
| F | 1 | Province | Female | 6.022E-04 | 0.1127 | 1.0465 | 0.6492 | 0.8815 | 0.9451 | 0.9740 | 0.9939 |
| F | 1 | Province | (Average) | 7.884E-04 | 0.1425 | 0.9799 | 0.6379 | 0.8758 | 0.9421 | 0.9723 | 0.9936 |
| G | (No Fourier term) | N/A | Male | 9.906E-04 | 0.1780 | 0.9437 | 0.6189 | 0.8652 | 0.9362 | 0.9686 | 0.9930 |
| G | (No Fourier term) | N/A | Female | 6.024E-04 | 0.1128 | 1.0471 | 0.6384 | 0.8727 | 0.9406 | 0.9709 | 0.9932 |
| G | (No Fourier term) | N/A | (Average) | 7.965E-04 | 0.1454 | 0.9954 | 0.6286 | 0.8689 | 0.9384 | 0.9697 | 0.9931 |

**Supplementary Table 6**: Out-of-sample predictive validity metrics comparing model estimates to observed data by province-year grouping. RMSE: root mean squared error. RSE: relative squared error.

| Model Specification | Number of Fourier terms | Seasonality fit separately by: | Sex | RMSE | RSE | 50% UI | 80% UI | 90% UI | 95% UI | 99% UI |
|---|---|---|---|---|---|---|---|---|---|---|
| A | 3 | Age, province | Male | 2.306E-05 | 0.3643 | 0.6703 | 0.9239 | 0.9725 | 0.9874 | 0.9983 |
| A | 3 | Age, province | Female | 2.513E-05 | 0.3477 | 0.6656 | 0.9125 | 0.9648 | 0.9856 | 0.9969 |
| A | 3 | Age, province | (Average) | 2.410E-05 | 0.3560 | 0.6680 | 0.9182 | 0.9686 | 0.9865 | 0.9976 |
| B | 2 | Age, province | Male | 2.205E-05 | 0.3332 | 0.7201 | 0.9455 | 0.9798 | 0.9925 | 0.9990 |
| B | 2 | Age, province | Female | 2.433E-05 | 0.3259 | 0.6975 | 0.9325 | 0.9766 | 0.9900 | 0.9986 |
| B | 2 | Age, province | (Average) | 2.319E-05 | 0.3296 | 0.7088 | 0.9390 | 0.9782 | 0.9912 | 0.9988 |
| C | 1 | Age, province | Male | 2.298E-05 | 0.3618 | 0.6647 | 0.9188 | 0.9689 | 0.9835 | 0.9971 |
| C | 1 | Age, province | Female | 2.589E-05 | 0.3691 | 0.6503 | 0.9030 | 0.9602 | 0.9818 | 0.9947 |
| C | 1 | Age, province | (Average) | 2.444E-05 | 0.3654 | 0.6575 | 0.9109 | 0.9646 | 0.9827 | 0.9959 |
| D | 3 | Province | Male | 2.223E-05 | 0.3384 | 0.7303 | 0.9477 | 0.9832 | 0.9946 | 0.9993 |
| D | 3 | Province | Female | 2.679E-05 | 0.3953 | 0.7140 | 0.9455 | 0.9837 | 0.9934 | 0.9990 |
| D | 3 | Province | (Average) | 2.451E-05 | 0.3669 | 0.7222 | 0.9466 | 0.9834 | 0.9940 | 0.9992 |
| E | 2 | Province | Male | 2.254E-05 | 0.3479 | 0.7062 | 0.9410 | 0.9786 | 0.9922 | 0.9995 |
| E | 2 | Province | Female | 2.517E-05 | 0.3488 | 0.7001 | 0.9409 | 0.9815 | 0.9922 | 0.9992 |
| E | 2 | Province | (Average) | 2.385E-05 | 0.3484 | 0.7031 | 0.9410 | 0.9800 | 0.9922 | 0.9993 |
| F | 1 | Province | Male | 2.320E-05 | 0.3687 | 0.6892 | 0.9283 | 0.9715 | 0.9866 | 0.9971 |
| F | 1 | Province | Female | 2.876E-05 | 0.4553 | 0.6754 | 0.9201 | 0.9698 | 0.9873 | 0.9976 |
| F | 1 | Province | (Average) | 2.598E-05 | 0.4120 | 0.6823 | 0.9242 | 0.9706 | 0.9869 | 0.9974 |
| G | (No Fourier term) | N/A | Male | 2.605E-05 | 0.4647 | 0.6112 | 0.8916 | 0.9545 | 0.9825 | 0.9952 |
| G | (No Fourier term) | N/A | Female | 3.044E-05 | 0.5101 | 0.5934 | 0.8748 | 0.9456 | 0.9755 | 0.9946 |
| G | (No Fourier term) | N/A | (Average) | 2.824E-05 | 0.4874 | 0.6023 | 0.8832 | 0.9500 | 0.9790 | 0.9949 |

Based on out-of-sample predictive validity metrics by province-year grouping, as summarized in Supplementary Table 6, we selected specification B as the model type that was used in the final analysis. This model specification had the lowest root mean squared error across both male and female runs, with slightly conservative empirical coverage of the 95% and 99% uncertainty intervals. Out-of-sample predictive validity metrics did not strongly differentiate between the model specifications that included any seasonality terms. However, the model specification that included no seasonality term performed worst in terms of both in-sample and out-of-sample predictive validity.

## Excess mortality time series estimation

When calculating a time series of excess mortality among small population groups, the cumulative effect of past mortality on the base population must be taken into account. This problem is most clearly illustrated under artificial conditions of a very high mortality rate. Imagine a population of 1,000 individuals that, under normal conditions, experiences a baseline mortality rate of 100 deaths per 1,000 person-weeks and predictably gains 100 new members at the end of each week. Under normal conditions, 100 individuals from the population would die and 100 new individuals would enter the population each week, resulting in a stable population week-to-week. Now, imagine an event that causes the mortality rate to increase to 900 deaths per 1,000 person-weeks. In the first week under these conditions, 900 individuals from the base population of 1,000 would die and 100 would be added, leading to a population of 200 entering the second week. In the second week, 180 of 200 individuals in the population would die. Although the standardized mortality ratio of this event compared to baseline is 9 across both weeks (900/100 and 180/20), an analysis that mistakenly assumed a starting population of 1,000 in the second week would dramatically underestimate this ratio as 1.8 (180/100).

This analysis accounted for the effects of excess mortality on subsequent population denominators throughout the study weeks beginning on February 26, 2020. Population denominators listed by Istat for January 1, 2020 were used as the starting denominators for this time series. For each predictive posterior draw and week, calculated excess deaths were subtracted from the base population used as the denominator in the subsequent week. The cumulative effect of this correction is to avoid understating the toll of excess mortality over the study period due to a reduction in the base population.

## Effect of mortality reductions on study findings

When considering age-specific excess mortality in Italy from 26 February 2020 through 26 May 2020, we identified 11 provinces where observed mortality for the 0-59 age group fell below the 95% uncertainty interval for baseline mortality, indicating significantly negative excess mortality. Among these provinces, the largest difference between observed and expected baseline deaths in the 0 to 59 age group is 29 fewer deaths (95% UI: 15 to 43 fewer deaths) in Caserta province, Campania. Additionally, five provinces were found to have significantly negative excess mortality for the 60 to 69 age group, and two provinces had significantly negative excess mortality for the 70 to 79 age group. Among the five unique provinces where significantly negative excess mortality was observed in older age groups, none were in the northern regions of Italy where most COVID-19 deaths were concentrated during March through May 2020, and which was the focus of our detailed analysis. These provinces and observed versus expected all-cause mortality totals are listed below in Supplementary Table 7.

**Supplementary Table 7**: Summary of province-age groupings with significant negative excess deaths during the study period. SMRs: standardized mortality ratios.

| Age group | Macroregion | Region | Province | Observed deaths | Expected baseline | Excess deaths | SMRs |
|---|---|---|---|---|---|---|---|
| 0 to 59 | Insular | Sicilia | Palermo | 223 | 248 (232 to 266) | -25 (-43 to -9) | 0.90 (0.84 to 0.96) |
| 0 to 59 | Insular | Sicilia | Trapani | 76 | 88 (80 to 97) | -12 (-21 to -4) | 0.86 (0.78 to 0.95) |
| 0 to 59 | Northeast | Friuli-Venezia Giulia | Pordenone | 46 | 51 (46 to 57) | -5 (-11 to 0) | 0.89 (0.80 to 1.00) |
| 0 to 59 | Northeast | Veneto | Belluno | 26 | 36 (32 to 40) | -10 (-14 to -6) | 0.73 (0.64 to 0.82) |
| 0 to 59 | Northeast | Veneto | Padova | 143 | 155 (145 to 166) | -12 (-23 to -2) | 0.92 (0.86 to 0.99) |
| 0 to 59 | Northeast | Veneto | Vicenza | 133 | 149 (139 to 160) | -16 (-27 to -6) | 0.89 (0.83 to 0.96) |
| 0 to 59 | South | Abruzzo | L'Aquila | 38 | 52 (47 to 58) | -14 (-20 to -9) | 0.73 (0.65 to 0.81) |
| 0 to 59 | South | Calabria | Crotone | 32 | 40 (36 to 46) | -8 (-14 to -4) | 0.79 (0.70 to 0.90) |
| 0 to 59 | South | Calabria | Vibo Valentia | 26 | 31 (27 to 36) | -5 (-10 to -1) | 0.83 (0.72 to 0.97) |
| 0 to 59 | South | Campania | Caserta | 186 | 215 (201 to 229) | -29 (-43 to -15) | 0.87 (0.81 to 0.92) |
| 0 to 59 | South | Molise | Campobasso | 30 | 37 (33 to 42) | -7 (-12 to -3) | 0.81 (0.72 to 0.91) |
| 60 to 69 | Central | Marche | Fermo | 33 | 43 (35 to 52) | -10 (-19 to -2) | 0.77 (0.64 to 0.95) |
| 60 to 69 | Central | Umbria | Terni | 42 | 56 (46 to 67) | -14 (-25 to -4) | 0.75 (0.62 to 0.92) |
| 60 to 69 | Insular | Sardegna | Nuoro | 54 | 79 (64 to 95) | -25 (-41 to -10) | 0.68 (0.57 to 0.84) |
| 60 to 69 | Insular | Sardegna | Sassari | 133 | 186 (155 to 219) | -53 (-86 to -22) | 0.71 (0.61 to 0.86) |
| 60 to 69 | Insular | Sicilia | Trapani | 83 | 106 (87 to 125) | -23 (-42 to -4) | 0.78 (0.66 to 0.95) |
| 70 to 79 | Insular | Sardegna | Nuoro | 101 | 143 (117 to 171) | -42 (-70 to -16) | 0.71 (0.59 to 0.86) |
| 70 to 79 | Insular | Sardegna | Sassari | 255 | 352 (289 to 416) | -97 (-161 to -34) | 0.72 (0.61 to 0.88) |

The total difference between observed and expected baseline deaths across these province-age groupings is a reduction of 409 deaths, of which 145 fewer deaths than expected occur in the 0 to 59 age group. These negative excess deaths were included in national estimates of age-specific and overall excess mortality. When these negative deaths are included, 51,647 of 53,245 (97.0%) excess deaths are attributed to the 60+ age groups. If all 18 significantly negative province-age groupings listed above are excluded from the calculation of total excess deaths, then 51,911 of 53,653 (96.8%) excess deaths are attributed to the 60+ age groups.

## Excess mortality results by province

Results from the excess mortality analysis from 26 February through 26 May 2020 are summarized by province in Supplementary Table 8.

**Supplementary Table 8**: Summary of excess mortality results by province. SMRs: standardized mortality ratios.

| Macroregion | Region | Province | Observed deaths | Expected baseline | Excess deaths | SMRs |
|---|---|---|---|---|---|---|
| Central | Lazio | Frosinone | 1,427 | 1,382 (1,142 to 1,615) | 45 (-188 to 285) | 1.03 (0.88 to 1.25) |
| Central | Lazio | Latina | 1,392 | 1,342 (1,117 to 1,564) | 50 (-172 to 275) | 1.04 (0.89 to 1.25) |
| Central | Lazio | Rieti | 515 | 463 (378 to 542) | 52 (-27 to 137) | 1.11 (0.95 to 1.36) |
| Central | Lazio | Roma | 10,548 | 10,183 (8,466 to 11,911) | 365 (-1,363 to 2,082) | 1.04 (0.89 to 1.25) |
| Central | Lazio | Viterbo | 909 | 942 (781 to 1,095) | -33 (-186 to 128) | 0.97 (0.83 to 1.16) |
| Central | Marche | Ancona | 1,601 | 1,289 (1,058 to 1,510) | 312 (91 to 543) | 1.24 (1.06 to 1.51) |
| Central | Marche | Ascoli Piceno | 618 | 593 (488 to 694) | 25 (-76 to 130) | 1.04 (0.89 to 1.27) |
| Central | Marche | Fermo | 613 | 507 (419 to 594) | 106 (19 to 194) | 1.21 (1.03 to 1.46) |
| Central | Marche | Macerata | 1,119 | 898 (739 to 1,056) | 221 (63 to 380) | 1.25 (1.06 to 1.51) |
| Central | Marche | Pesaro e Urbino | 1,725 | 959 (792 to 1,122) | 766 (603 to 933) | 1.80 (1.54 to 2.18) |
| Central | Toscana | Arezzo | 1,051 | 983 (812 to 1,151) | 68 (-100 to 239) | 1.07 (0.91 to 1.29) |
| Central | Toscana | Firenze | 3,242 | 2,723 (2,247 to 3,189) | 519 (53 to 995) | 1.19 (1.02 to 1.44) |
| Central | Toscana | Grosseto | 767 | 696 (575 to 813) | 71 (-46 to 192) | 1.10 (0.94 to 1.33) |
| Central | Toscana | Livorno | 1,174 | 1,006 (827 to 1,179) | 168 (-5 to 347) | 1.17 (1.00 to 1.42) |
| Central | Toscana | Lucca | 1,289 | 1,165 (967 to 1,358) | 124 (-69 to 322) | 1.11 (0.95 to 1.33) |
| Central | Toscana | Massa Carrara | 829 | 642 (529 to 754) | 187 (75 to 300) | 1.29 (1.10 to 1.57) |
| Central | Toscana | Pisa | 1,325 | 1,144 (939 to 1,339) | 181 (-14 to 386) | 1.16 (0.99 to 1.41) |
| Central | Toscana | Pistoia | 915 | 806 (669 to 946) | 109 (-31 to 246) | 1.13 (0.97 to 1.37) |
| Central | Toscana | Prato | 652 | 631 (522 to 740) | 21 (-88 to 130) | 1.03 (0.88 to 1.25) |
| Central | Toscana | Siena | 825 | 796 (652 to 936) | 29 (-111 to 173) | 1.04 (0.88 to 1.27) |
| Central | Umbria | Perugia | 1,903 | 1,812 (1,503 to 2,122) | 91 (-219 to 400) | 1.05 (0.90 to 1.27) |
| Central | Umbria | Terni | 777 | 715 (586 to 835) | 62 (-58 to 191) | 1.09 (0.93 to 1.33) |
| Insular | Sardegna | Cagliari | 1,013 | 800 (670 to 929) | 213 (84 to 343) | 1.27 (1.09 to 1.51) |
| Insular | Sardegna | Nuoro | 598 | 725 (603 to 849) | -127 (-251 to -5) | 0.83 (0.70 to 0.99) |
| Insular | Sardegna | Oristano | 531 | 483 (397 to 566) | 48 (-35 to 134) | 1.10 (0.94 to 1.34) |
| Insular | Sardegna | Sassari | 1,343 | 1,532 (1,270 to 1,784) | -189 (-441 to 73) | 0.88 (0.75 to 1.06) |
| Insular | Sardegna | Sud Sardegna | 1,070 | 954 (793 to 1,118) | 116 (-48 to 277) | 1.12 (0.96 to 1.35) |
| Insular | Sicilia | Agrigento | 1,274 | 1,259 (1,043 to 1,473) | 15 (-199 to 231) | 1.01 (0.87 to 1.22) |
| Insular | Sicilia | Caltanissetta | 769 | 740 (611 to 866) | 29 (-97 to 158) | 1.04 (0.89 to 1.26) |
| Insular | Sicilia | Catania | 2,639 | 2,711 (2,259 to 3,156) | -72 (-517 to 380) | 0.97 (0.84 to 1.17) |
| Insular | Sicilia | Enna | 572 | 523 (428 to 610) | 49 (-38 to 144) | 1.09 (0.94 to 1.34) |
| Insular | Sicilia | Messina | 1,948 | 1,828 (1,518 to 2,138) | 120 (-190 to 430) | 1.07 (0.91 to 1.28) |
| Insular | Sicilia | Palermo | 3,305 | 3,222 (2,681 to 3,762) | 83 (-457 to 624) | 1.03 (0.88 to 1.23) |
| Insular | Sicilia | Ragusa | 832 | 800 (671 to 931) | 32 (-99 to 161) | 1.04 (0.89 to 1.24) |
| Insular | Sicilia | Siracusa | 1,139 | 1,021 (857 to 1,193) | 118 (-54 to 282) | 1.12 (0.95 to 1.33) |

| Macroregion | Region | Province | Observed deaths | Expected baseline | Excess deaths | SMRs |
|---|---|---|---|---|---|---|
| Insular | Sicilia | Trapani | 1,210 | 1,200 (999 to 1,398) | 10 (-188 to 211) | 1.01 (0.87 to 1.21) |
| Northeast | Emilia-Romagna | Bologna | 3,670 | 2,779 (2,300 to 3,252) | 891 (418 to 1,370) | 1.32 (1.13 to 1.60) |
| Northeast | Emilia-Romagna | Ferrara | 1,324 | 1,148 (946 to 1,353) | 176 (-29 to 378) | 1.15 (0.98 to 1.40) |
| Northeast | Emilia-Romagna | Forli'-Cesena | 1,361 | 1,059 (877 to 1,244) | 302 (117 to 484) | 1.28 (1.09 to 1.55) |
| Northeast | Emilia-Romagna | Modena | 2,340 | 1,754 (1,453 to 2,056) | 586 (284 to 887) | 1.33 (1.14 to 1.61) |
| Northeast | Emilia-Romagna | Parma | 2,700 | 1,190 (983 to 1,398) | 1,510 (1,302 to 1,717) | 2.27 (1.93 to 2.75) |
| Northeast | Emilia-Romagna | Piacenza | 2,209 | 857 (704 to 1,002) | 1,352 (1,207 to 1,505) | 2.58 (2.20 to 3.14) |
| Northeast | Emilia-Romagna | Ravenna | 1,265 | 1,118 (925 to 1,308) | 147 (-43 to 340) | 1.13 (0.97 to 1.37) |
| Northeast | Emilia-Romagna | Reggio Emilia | 2,006 | 1,324 (1,097 to 1,544) | 682 (462 to 909) | 1.51 (1.30 to 1.83) |
| Northeast | Emilia-Romagna | Rimini | 1,175 | 807 (662 to 944) | 368 (231 to 513) | 1.46 (1.24 to 1.77) |
| Northeast | Friuli-Venezia Giulia | Gorizia | 465 | 440 (361 to 513) | 25 (-48 to 104) | 1.06 (0.91 to 1.29) |
| Northeast | Friuli-Venezia Giulia | Pordenone | 908 | 792 (656 to 926) | 116 (-18 to 252) | 1.15 (0.98 to 1.38) |
| Northeast | Friuli-Venezia Giulia | Trieste | 994 | 800 (666 to 931) | 194 (63 to 328) | 1.24 (1.07 to 1.49) |
| Northeast | Friuli-Venezia Giulia | Udine | 1,675 | 1,589 (1,315 to 1,852) | 86 (-177 to 360) | 1.05 (0.90 to 1.27) |
| Northeast | Trentino-Alto Adige | Bolzano | 1,608 | 1,098 (916 to 1,284) | 510 (324 to 692) | 1.46 (1.25 to 1.76) |
| Northeast | Trentino-Alto Adige | Trento | 1,973 | 1,265 (1,048 to 1,485) | 708 (488 to 925) | 1.56 (1.33 to 1.88) |
| Northeast | Veneto | Belluno | 726 | 627 (517 to 732) | 99 (-6 to 209) | 1.16 (0.99 to 1.40) |
| Northeast | Veneto | Padova | 2,516 | 2,247 (1,858 to 2,619) | 269 (-103 to 658) | 1.12 (0.96 to 1.35) |
| Northeast | Veneto | Rovigo | 871 | 743 (615 to 868) | 128 (3 to 256) | 1.17 (1.00 to 1.42) |
| Northeast | Veneto | Treviso | 2,505 | 2,060 (1,714 to 2,422) | 445 (83 to 791) | 1.22 (1.03 to 1.46) |
| Northeast | Veneto | Venezia | 2,712 | 2,243 (1,860 to 2,628) | 469 (84 to 852) | 1.21 (1.03 to 1.46) |
| Northeast | Veneto | Verona | 2,893 | 2,200 (1,821 to 2,569) | 693 (324 to 1,072) | 1.32 (1.13 to 1.59) |
| Northeast | Veneto | Vicenza | 2,404 | 2,020 (1,681 to 2,357) | 384 (47 to 723) | 1.19 (1.02 to 1.43) |
| Northwest | Liguria | Genova | 4,354 | 2,841 (2,349 to 3,326) | 1,513 (1,028 to 2,005) | 1.53 (1.31 to 1.85) |
| Northwest | Liguria | Imperia | 1,048 | 725 (597 to 851) | 323 (197 to 451) | 1.44 (1.23 to 1.76) |
| Northwest | Liguria | La Spezia | 988 | 708 (581 to 834) | 280 (154 to 407) | 1.40 (1.19 to 1.70) |
| Northwest | Liguria | Savona | 1,283 | 951 (783 to 1,122) | 332 (161 to 500) | 1.35 (1.14 to 1.64) |
| Northwest | Lombardia | Bergamo | 8,702 | 2,453 (2,049 to 2,863) | 6,249 (5,839 to 6,653) | 3.55 (3.04 to 4.25) |
| Northwest | Lombardia | Brescia | 7,474 | 2,885 (2,390 to 3,376) | 4,589 (4,098 to 5,084) | 2.59 (2.21 to 3.13) |
| Northwest | Lombardia | Como | 2,340 | 1,498 (1,252 to 1,757) | 842 (583 to 1,088) | 1.56 (1.33 to 1.87) |
| Northwest | Lombardia | Cremona | 3,156 | 988 (819 to 1,161) | 2,168 (1,995 to 2,337) | 3.19 (2.72 to 3.85) |
| Northwest | Lombardia | Lecco | 1,722 | 795 (659 to 928) | 927 (794 to 1,063) | 2.17 (1.86 to 2.61) |
| Northwest | Lombardia | Lodi | 1,538 | 559 (463 to 654) | 979 (884 to 1,075) | 2.75 (2.35 to 3.32) |
| Northwest | Lombardia | Mantova | 2,065 | 1,100 (910 to 1,289) | 965 (776 to 1,155) | 1.88 (1.60 to 2.27) |
| Northwest | Lombardia | Milano | 13,749 | 7,557 (6,273 to 8,873) | 6,192 (4,876 to 7,476) | 1.82 (1.55 to 2.19) |
| Northwest | Lombardia | Monza e della Brianza | 3,331 | 1,919 (1,589 to 2,243) | 1,412 (1,088 to 1,742) | 1.74 (1.49 to 2.10) |
| Northwest | Lombardia | Pavia | 3,426 | 1,636 (1,357 to 1,917) | 1,790 (1,509 to 2,069) | 2.09 (1.79 to 2.53) |
| Northwest | Lombardia | Sondrio | 834 | 496 (410 to 578) | 338 (256 to 424) | 1.68 (1.44 to 2.03) |
| Northwest | Lombardia | Varese | 3,187 | 2,236 (1,854 to 2,611) | 951 (576 to 1,333) | 1.43 (1.22 to 1.72) |
| Northwest | Piemonte | Alessandria | 2,511 | 1,440 (1,186 to 1,689) | 1,071 (822 to 1,325) | 1.74 (1.49 to 2.12) |

| Macroregion | Region | Province | Observed deaths | Expected baseline | Excess deaths | SMRs |
|---|---|---|---|---|---|---|
| Northwest | Piemonte | Asti | 1,032 | 692 (569 to 808) | 340 (224 to 463) | 1.49 (1.28 to 1.81) |
| Northwest | Piemonte | Biella | 987 | 586 (483 to 688) | 401 (299 to 504) | 1.69 (1.43 to 2.05) |
| Northwest | Piemonte | Cuneo | 2,212 | 1,705 (1,407 to 1,996) | 507 (216 to 805) | 1.30 (1.11 to 1.57) |
| Northwest | Piemonte | Novara | 1,631 | 1,003 (827 to 1,167) | 628 (464 to 804) | 1.63 (1.40 to 1.97) |
| Northwest | Piemonte | Torino | 9,285 | 6,221 (5,162 to 7,300) | 3,064 (1,985 to 4,123) | 1.49 (1.27 to 1.80) |
| Northwest | Piemonte | Verbano-Cusio-Ossola | 690 | 499 (413 to 584) | 191 (106 to 277) | 1.38 (1.18 to 1.67) |
| Northwest | Piemonte | Vercelli | 949 | 581 (478 to 684) | 368 (265 to 471) | 1.63 (1.39 to 1.98) |
| Northwest | Valle d'Aosta | Aosta | 519 | 363 (300 to 427) | 156 (92 to 219) | 1.43 (1.21 to 1.73) |
| South | Abruzzo | Chieti | 1,285 | 1,142 (945 to 1,336) | 143 (-51 to 340) | 1.13 (0.96 to 1.36) |
| South | Abruzzo | L'Aquila | 869 | 856 (704 to 998) | 13 (-129 to 165) | 1.01 (0.87 to 1.24) |
| South | Abruzzo | Pescara | 1,079 | 837 (697 to 985) | 242 (94 to 382) | 1.29 (1.10 to 1.55) |
| South | Abruzzo | Teramo | 962 | 826 (684 to 966) | 136 (-4 to 278) | 1.16 (1.00 to 1.41) |
| South | Basilicata | Matera | 545 | 507 (420 to 589) | 38 (-44 to 125) | 1.08 (0.93 to 1.30) |
| South | Basilicata | Potenza | 1,148 | 1,077 (891 to 1,257) | 71 (-109 to 257) | 1.07 (0.91 to 1.29) |
| South | Calabria | Catanzaro | 940 | 909 (754 to 1,062) | 31 (-122 to 186) | 1.03 (0.89 to 1.25) |
| South | Calabria | Cosenza | 2,019 | 1,859 (1,544 to 2,170) | 160 (-151 to 475) | 1.09 (0.93 to 1.31) |
| South | Calabria | Crotone | 462 | 411 (341 to 475) | 51 (-13 to 121) | 1.12 (0.97 to 1.36) |
| South | Calabria | Reggio di Calabria | 1,555 | 1,405 (1,169 to 1,640) | 150 (-85 to 386) | 1.11 (0.95 to 1.33) |
| South | Calabria | Vibo Valentia | 415 | 401 (333 to 468) | 14 (-53 to 82) | 1.04 (0.89 to 1.25) |
| South | Campania | Avellino | 1,319 | 1,171 (973 to 1,368) | 148 (-49 to 346) | 1.13 (0.96 to 1.36) |
| South | Campania | Benevento | 798 | 787 (651 to 926) | 11 (-128 to 147) | 1.01 (0.86 to 1.23) |
| South | Campania | Caserta | 1,972 | 1,995 (1,677 to 2,318) | -23 (-346 to 295) | 0.99 (0.85 to 1.18) |
| South | Campania | Napoli | 6,870 | 6,732 (5,659 to 7,807) | 138 (-937 to 1,211) | 1.02 (0.88 to 1.21) |
| South | Campania | Salerno | 2,767 | 2,813 (2,346 to 3,279) | -46 (-512 to 421) | 0.98 (0.84 to 1.18) |
| South | Molise | Campobasso | 703 | 658 (542 to 769) | 45 (-66 to 161) | 1.07 (0.91 to 1.30) |
| South | Molise | Isernia | 286 | 279 (230 to 326) | 7 (-40 to 56) | 1.02 (0.88 to 1.24) |
| South | Puglia | Bari | 3,186 | 2,819 (2,352 to 3,309) | 367 (-123 to 834) | 1.13 (0.96 to 1.35) |
| South | Puglia | Barletta-Andria-Trani | 948 | 832 (700 to 963) | 116 (-15 to 248) | 1.14 (0.98 to 1.35) |
| South | Puglia | Brindisi | 1,179 | 1,007 (834 to 1,179) | 172 (0 to 345) | 1.17 (1.00 to 1.41) |
| South | Puglia | Foggia | 1,803 | 1,516 (1,266 to 1,761) | 287 (42 to 537) | 1.19 (1.02 to 1.42) |
| South | Puglia | Lecce | 2,275 | 2,108 (1,750 to 2,461) | 167 (-186 to 525) | 1.08 (0.92 to 1.30) |
| South | Puglia | Taranto | 1,499 | 1,399 (1,169 to 1,634) | 100 (-135 to 330) | 1.07 (0.92 to 1.28) |

## Excess mortality results by province and age group

Results from the excess mortality analysis from 26 February through 26 May 2020 are summarized by province and age group in Supplementary Table 9.

**Supplementary Table 9**: Summary of excess mortality results by province and age group. SMRs: standardized mortality ratios.

| Macroregion | Region | Province | Age group | Observed deaths | Expected baseline | Excess deaths | SMRs |
|---|---|---|---|---|---|---|---|
| Central | Lazio | Frosinone | 0 to 59 | 102 | 94 (87 to 103) | 8 (-1 to 15) | 1.08 (0.99 to 1.17) |
| Central | Lazio | Frosinone | 60 to 69 | 148 | 136 (113 to 161) | 12 (-13 to 35) | 1.09 (0.92 to 1.31) |
| Central | Lazio | Frosinone | 70 to 79 | 248 | 268 (221 to 316) | -20 (-68 to 27) | 0.93 (0.78 to 1.12) |
| Central | Lazio | Frosinone | 80 to 89 | 538 | 543 (426 to 649) | -5 (-111 to 112) | 0.99 (0.83 to 1.26) |
| Central | Lazio | Frosinone | 90+ | 391 | 341 (270 to 411) | 50 (-20 to 121) | 1.15 (0.95 to 1.45) |
| Central | Lazio | Latina | 0 to 59 | 110 | 107 (99 to 117) | 3 (-7 to 11) | 1.02 (0.94 to 1.11) |
| Central | Lazio | Latina | 60 to 69 | 132 | 132 (110 to 157) | 0 (-25 to 22) | 1.00 (0.84 to 1.20) |
| Central | Lazio | Latina | 70 to 79 | 286 | 286 (235 to 340) | 0 (-54 to 51) | 1.00 (0.84 to 1.22) |
| Central | Lazio | Latina | 80 to 89 | 535 | 528 (420 to 638) | 7 (-103 to 115) | 1.01 (0.84 to 1.27) |
| Central | Lazio | Latina | 90+ | 329 | 289 (229 to 345) | 40 (-16 to 100) | 1.14 (0.95 to 1.44) |
| Central | Lazio | Rieti | 0 to 59 | 48 | 25 (22 to 29) | 23 (19 to 26) | 1.91 (1.68 to 2.19) |
| Central | Lazio | Rieti | 60 to 69 | 44 | 42 (34 to 50) | 2 (-6 to 10) | 1.05 (0.88 to 1.29) |
| Central | Lazio | Rieti | 70 to 79 | 87 | 85 (70 to 102) | 2 (-15 to 17) | 1.02 (0.85 to 1.24) |
| Central | Lazio | Rieti | 80 to 89 | 191 | 184 (144 to 223) | 7 (-32 to 47) | 1.04 (0.86 to 1.32) |
| Central | Lazio | Rieti | 90+ | 145 | 126 (100 to 153) | 19 (-8 to 45) | 1.15 (0.95 to 1.45) |
| Central | Lazio | Roma | 0 to 59 | 779 | 775 (743 to 807) | 4 (-28 to 36) | 1.01 (0.97 to 1.05) |
| Central | Lazio | Roma | 60 to 69 | 1,007 | 986 (833 to 1,154) | 21 (-147 to 174) | 1.02 (0.87 to 1.21) |
| Central | Lazio | Roma | 70 to 79 | 2,212 | 2,021 (1,679 to 2,379) | 191 (-167 to 533) | 1.09 (0.93 to 1.32) |
| Central | Lazio | Roma | 80 to 89 | 4,063 | 4,029 (3,184 to 4,847) | 34 (-784 to 879) | 1.01 (0.84 to 1.28) |
| Central | Lazio | Roma | 90+ | 2,487 | 2,373 (1,894 to 2,851) | 114 (-364 to 593) | 1.05 (0.87 to 1.31) |
| Central | Lazio | Viterbo | 0 to 59 | 57 | 62 (56 to 69) | -5 (-12 to 1) | 0.92 (0.83 to 1.02) |
| Central | Lazio | Viterbo | 60 to 69 | 93 | 85 (71 to 101) | 8 (-8 to 22) | 1.09 (0.92 to 1.31) |
| Central | Lazio | Viterbo | 70 to 79 | 176 | 182 (149 to 216) | -6 (-40 to 27) | 0.97 (0.82 to 1.18) |
| Central | Lazio | Viterbo | 80 to 89 | 335 | 386 (307 to 464) | -51 (-129 to 28) | 0.87 (0.72 to 1.09) |
| Central | Lazio | Viterbo | 90+ | 248 | 226 (180 to 271) | 22 (-23 to 68) | 1.10 (0.91 to 1.38) |
| Central | Marche | Ancona | 0 to 59 | 91 | 74 (66 to 81) | 17 (10 to 25) | 1.24 (1.12 to 1.37) |
| Central | Marche | Ancona | 60 to 69 | 108 | 106 (88 to 126) | 2 (-18 to 20) | 1.02 (0.85 to 1.23) |
| Central | Marche | Ancona | 70 to 79 | 265 | 210 (174 to 250) | 55 (15 to 91) | 1.26 (1.06 to 1.53) |
| Central | Marche | Ancona | 80 to 89 | 655 | 519 (407 to 622) | 136 (33 to 248) | 1.26 (1.05 to 1.61) |
| Central | Marche | Ancona | 90+ | 482 | 380 (302 to 458) | 102 (24 to 180) | 1.27 (1.05 to 1.60) |
| Central | Marche | Ascoli Piceno | 0 to 59 | 35 | 33 (29 to 38) | 2 (-3 to 6) | 1.05 (0.93 to 1.19) |
| Central | Marche | Ascoli Piceno | 60 to 69 | 45 | 45 (36 to 53) | 0 (-8 to 9) | 1.01 (0.84 to 1.24) |
| Central | Marche | Ascoli Piceno | 70 to 79 | 101 | 97 (80 to 116) | 4 (-15 to 21) | 1.04 (0.87 to 1.26) |
| Central | Marche | Ascoli Piceno | 80 to 89 | 243 | 250 (198 to 300) | -7 (-57 to 45) | 0.97 (0.81 to 1.22) |
| Central | Marche | Ascoli Piceno | 90+ | 194 | 169 (134 to 204) | 25 (-10 to 60) | 1.15 (0.95 to 1.45) |
| Central | Marche | Fermo | 0 to 59 | 32 | 28 (24 to 32) | 4 (0 to 8) | 1.14 (1.00 to 1.31) |

| Macroregion | Region | Province | Age group | Observed deaths | Expected baseline | Excess deaths | SMRs |
|---|---|---|---|---|---|---|---|
| Central | Marche | Fermo | 60 to 69 | 33 | 43 (35 to 52) | -10 (-19 to -2) | 0.77 (0.64 to 0.95) |
| Central | Marche | Fermo | 70 to 79 | 104 | 82 (68 to 99) | 22 (5 to 36) | 1.26 (1.05 to 1.53) |
| Central | Marche | Fermo | 80 to 89 | 261 | 220 (173 to 265) | 41 (-4 to 88) | 1.19 (0.99 to 1.51) |
| Central | Marche | Fermo | 90+ | 183 | 133 (106 to 163) | 50 (20 to 77) | 1.37 (1.12 to 1.72) |
| Central | Marche | Macerata | 0 to 59 | 66 | 48 (43 to 53) | 18 (13 to 23) | 1.37 (1.23 to 1.53) |
| Central | Marche | Macerata | 60 to 69 | 71 | 65 (53 to 76) | 6 (-5 to 18) | 1.10 (0.93 to 1.33) |
| Central | Marche | Macerata | 70 to 79 | 203 | 145 (120 to 173) | 58 (30 to 83) | 1.40 (1.17 to 1.70) |
| Central | Marche | Macerata | 80 to 89 | 434 | 379 (299 to 460) | 55 (-26 to 135) | 1.15 (0.94 to 1.45) |
| Central | Marche | Macerata | 90+ | 345 | 261 (208 to 315) | 84 (30 to 137) | 1.32 (1.09 to 1.66) |
| Central | Marche | Pesaro e Urbino | 0 to 59 | 85 | 53 (47 to 59) | 32 (26 to 38) | 1.61 (1.44 to 1.80) |
| Central | Marche | Pesaro e Urbino | 60 to 69 | 133 | 69 (57 to 82) | 64 (51 to 76) | 1.92 (1.61 to 2.31) |
| Central | Marche | Pesaro e Urbino | 70 to 79 | 315 | 160 (131 to 189) | 155 (126 to 184) | 1.97 (1.67 to 2.40) |
| Central | Marche | Pesaro e Urbino | 80 to 89 | 686 | 386 (305 to 463) | 300 (223 to 381) | 1.78 (1.48 to 2.25) |
| Central | Marche | Pesaro e Urbino | 90+ | 506 | 291 (232 to 352) | 215 (154 to 274) | 1.74 (1.44 to 2.18) |
| Central | Toscana | Arezzo | 0 to 59 | 64 | 57 (51 to 63) | 7 (1 to 13) | 1.12 (1.02 to 1.25) |
| Central | Toscana | Arezzo | 60 to 69 | 92 | 75 (62 to 89) | 17 (3 to 30) | 1.22 (1.03 to 1.47) |
| Central | Toscana | Arezzo | 70 to 79 | 157 | 177 (144 to 211) | -20 (-54 to 13) | 0.89 (0.74 to 1.09) |
| Central | Toscana | Arezzo | 80 to 89 | 399 | 395 (312 to 476) | 4 (-77 to 87) | 1.01 (0.84 to 1.28) |
| Central | Toscana | Arezzo | 90+ | 339 | 279 (221 to 335) | 60 (4 to 118) | 1.22 (1.01 to 1.53) |
| Central | Toscana | Firenze | 0 to 59 | 166 | 145 (136 to 155) | 21 (11 to 30) | 1.14 (1.07 to 1.22) |
| Central | Toscana | Firenze | 60 to 69 | 227 | 196 (164 to 230) | 31 (-3 to 63) | 1.16 (0.99 to 1.38) |
| Central | Toscana | Firenze | 70 to 79 | 546 | 467 (383 to 547) | 79 (-1 to 163) | 1.17 (1.00 to 1.43) |
| Central | Toscana | Firenze | 80 to 89 | 1,309 | 1,102 (866 to 1,327) | 207 (-18 to 443) | 1.19 (0.99 to 1.51) |
| Central | Toscana | Firenze | 90+ | 994 | 812 (645 to 973) | 182 (21 to 349) | 1.22 (1.02 to 1.54) |
| Central | Toscana | Grosseto | 0 to 59 | 46 | 36 (31 to 40) | 10 (6 to 15) | 1.29 (1.14 to 1.46) |
| Central | Toscana | Grosseto | 60 to 69 | 51 | 54 (45 to 66) | -3 (-15 to 6) | 0.94 (0.78 to 1.14) |
| Central | Toscana | Grosseto | 70 to 79 | 144 | 130 (107 to 155) | 14 (-11 to 37) | 1.11 (0.93 to 1.35) |
| Central | Toscana | Grosseto | 80 to 89 | 294 | 285 (225 to 345) | 9 (-51 to 69) | 1.03 (0.85 to 1.30) |
| Central | Toscana | Grosseto | 90+ | 232 | 191 (150 to 232) | 41 (0 to 82) | 1.22 (1.00 to 1.55) |
| Central | Toscana | Livorno | 0 to 59 | 72 | 59 (53 to 65) | 13 (7 to 19) | 1.22 (1.10 to 1.37) |
| Central | Toscana | Livorno | 60 to 69 | 93 | 78 (65 to 94) | 15 (-1 to 28) | 1.19 (0.99 to 1.44) |
| Central | Toscana | Livorno | 70 to 79 | 235 | 205 (170 to 242) | 30 (-7 to 65) | 1.15 (0.97 to 1.38) |
| Central | Toscana | Livorno | 80 to 89 | 436 | 404 (320 to 485) | 32 (-49 to 116) | 1.08 (0.90 to 1.36) |
| Central | Toscana | Livorno | 90+ | 338 | 260 (204 to 314) | 78 (24 to 134) | 1.30 (1.08 to 1.66) |
| Central | Toscana | Lucca | 0 to 59 | 62 | 67 (61 to 74) | -5 (-12 to 1) | 0.93 (0.84 to 1.02) |
| Central | Toscana | Lucca | 60 to 69 | 98 | 94 (78 to 113) | 4 (-15 to 20) | 1.04 (0.87 to 1.25) |
| Central | Toscana | Lucca | 70 to 79 | 271 | 218 (180 to 260) | 53 (11 to 91) | 1.24 (1.04 to 1.51) |
| Central | Toscana | Lucca | 80 to 89 | 523 | 470 (371 to 563) | 53 (-40 to 152) | 1.11 (0.93 to 1.41) |
| Central | Toscana | Lucca | 90+ | 335 | 315 (249 to 382) | 20 (-47 to 86) | 1.06 (0.88 to 1.34) |
| Central | Toscana | Massa Carrara | 0 to 59 | 46 | 36 (32 to 41) | 10 (5 to 14) | 1.27 (1.13 to 1.45) |
| Central | Toscana | Massa Carrara | 60 to 69 | 73 | 51 (42 to 62) | 22 (11 to 31) | 1.42 (1.18 to 1.72) |
| Central | Toscana | Massa Carrara | 70 to 79 | 144 | 121 (98 to 144) | 23 (0 to 46) | 1.19 (1.00 to 1.47) |
| Central | Toscana | Massa Carrara | 80 to 89 | 341 | 251 (198 to 304) | 90 (37 to 143) | 1.36 (1.12 to 1.72) |
| Central | Toscana | Massa Carrara | 90+ | 225 | 182 (145 to 220) | 43 (5 to 80) | 1.23 (1.02 to 1.55) |

| Macroregion | Region | Province | Age group | Observed deaths | Expected baseline | Excess deaths | SMRs |
|---|---|---|---|---|---|---|---|
| Central | Toscana | Pisa | 0 to 59 | 80 | 71 (64 to 78) | 9 (2 to 16) | 1.13 (1.03 to 1.25) |
| Central | Toscana | Pisa | 60 to 69 | 85 | 93 (78 to 111) | -8 (-26 to 7) | 0.91 (0.77 to 1.09) |
| Central | Toscana | Pisa | 70 to 79 | 265 | 210 (172 to 249) | 55 (16 to 93) | 1.26 (1.06 to 1.54) |
| Central | Toscana | Pisa | 80 to 89 | 531 | 457 (361 to 549) | 74 (-18 to 170) | 1.16 (0.97 to 1.47) |
| Central | Toscana | Pisa | 90+ | 364 | 313 (247 to 377) | 51 (-13 to 117) | 1.16 (0.97 to 1.47) |
| Central | Toscana | Pistoia | 0 to 59 | 62 | 49 (44 to 54) | 13 (8 to 18) | 1.28 (1.15 to 1.42) |
| Central | Toscana | Pistoia | 60 to 69 | 82 | 65 (54 to 76) | 17 (6 to 28) | 1.27 (1.07 to 1.53) |
| Central | Toscana | Pistoia | 70 to 79 | 174 | 145 (119 to 173) | 29 (1 to 55) | 1.20 (1.01 to 1.46) |
| Central | Toscana | Pistoia | 80 to 89 | 356 | 329 (262 to 395) | 27 (-39 to 94) | 1.08 (0.90 to 1.36) |
| Central | Toscana | Pistoia | 90+ | 241 | 218 (173 to 264) | 23 (-23 to 68) | 1.10 (0.91 to 1.39) |
| Central | Toscana | Prato | 0 to 59 | 39 | 41 (36 to 46) | -2 (-7 to 3) | 0.96 (0.85 to 1.09) |
| Central | Toscana | Prato | 60 to 69 | 59 | 48 (40 to 58) | 11 (1 to 19) | 1.22 (1.01 to 1.49) |
| Central | Toscana | Prato | 70 to 79 | 112 | 113 (92 to 134) | -1 (-22 to 20) | 0.99 (0.83 to 1.21) |
| Central | Toscana | Prato | 80 to 89 | 265 | 251 (197 to 303) | 14 (-38 to 68) | 1.06 (0.87 to 1.35) |
| Central | Toscana | Prato | 90+ | 177 | 179 (142 to 216) | -2 (-39 to 35) | 0.99 (0.82 to 1.25) |
| Central | Toscana | Siena | 0 to 59 | 40 | 39 (35 to 44) | 1 (-4 to 5) | 1.01 (0.90 to 1.14) |
| Central | Toscana | Siena | 60 to 69 | 66 | 54 (44 to 64) | 12 (2 to 22) | 1.22 (1.03 to 1.49) |
| Central | Toscana | Siena | 70 to 79 | 141 | 135 (111 to 160) | 6 (-19 to 30) | 1.04 (0.88 to 1.27) |
| Central | Toscana | Siena | 80 to 89 | 324 | 316 (251 to 385) | 8 (-61 to 73) | 1.02 (0.84 to 1.29) |
| Central | Toscana | Siena | 90+ | 254 | 251 (199 to 303) | 3 (-49 to 55) | 1.01 (0.84 to 1.28) |
| Central | Umbria | Perugia | 0 to 59 | 109 | 105 (97 to 113) | 4 (-4 to 12) | 1.04 (0.96 to 1.13) |
| Central | Umbria | Perugia | 60 to 69 | 132 | 133 (112 to 157) | -1 (-25 to 20) | 0.99 (0.84 to 1.18) |
| Central | Umbria | Perugia | 70 to 79 | 305 | 315 (260 to 374) | -10 (-69 to 45) | 0.97 (0.82 to 1.17) |
| Central | Umbria | Perugia | 80 to 89 | 711 | 737 (589 to 887) | -26 (-176 to 122) | 0.96 (0.80 to 1.21) |
| Central | Umbria | Perugia | 90+ | 646 | 522 (415 to 628) | 124 (18 to 231) | 1.24 (1.03 to 1.56) |
| Central | Umbria | Terni | 0 to 59 | 48 | 40 (36 to 45) | 8 (3 to 12) | 1.21 (1.07 to 1.35) |
| Central | Umbria | Terni | 60 to 69 | 42 | 56 (46 to 67) | -14 (-25 to -4) | 0.75 (0.62 to 0.92) |
| Central | Umbria | Terni | 70 to 79 | 149 | 129 (106 to 153) | 20 (-4 to 43) | 1.15 (0.97 to 1.41) |
| Central | Umbria | Terni | 80 to 89 | 271 | 294 (233 to 354) | -23 (-83 to 38) | 0.92 (0.76 to 1.16) |
| Central | Umbria | Terni | 90+ | 267 | 196 (155 to 235) | 71 (32 to 112) | 1.36 (1.13 to 1.72) |
| Insular | Sardegna | Cagliari | 0 to 59 | 94 | 80 (72 to 88) | 14 (6 to 22) | 1.18 (1.07 to 1.31) |
| Insular | Sardegna | Cagliari | 60 to 69 | 116 | 85 (71 to 101) | 31 (15 to 45) | 1.37 (1.15 to 1.64) |
| Insular | Sardegna | Cagliari | 70 to 79 | 206 | 152 (125 to 181) | 54 (25 to 81) | 1.35 (1.14 to 1.65) |
| Insular | Sardegna | Cagliari | 80 to 89 | 336 | 274 (216 to 333) | 62 (3 to 120) | 1.23 (1.01 to 1.55) |
| Insular | Sardegna | Cagliari | 90+ | 261 | 209 (165 to 254) | 52 (7 to 96) | 1.25 (1.03 to 1.58) |
| Insular | Sardegna | Nuoro | 0 to 59 | 48 | 51 (45 to 58) | -3 (-10 to 3) | 0.94 (0.83 to 1.08) |
| Insular | Sardegna | Nuoro | 60 to 69 | 54 | 79 (64 to 95) | -25 (-41 to -10) | 0.68 (0.57 to 0.84) |
| Insular | Sardegna | Nuoro | 70 to 79 | 101 | 143 (117 to 171) | -42 (-70 to -16) | 0.71 (0.59 to 0.86) |
| Insular | Sardegna | Nuoro | 80 to 89 | 235 | 283 (222 to 344) | -48 (-109 to 13) | 0.83 (0.68 to 1.06) |
| Insular | Sardegna | Nuoro | 90+ | 160 | 169 (134 to 203) | -9 (-43 to 26) | 0.95 (0.79 to 1.19) |
| Insular | Sardegna | Oristano | 0 to 59 | 40 | 30 (26 to 34) | 10 (6 to 14) | 1.34 (1.17 to 1.55) |
| Insular | Sardegna | Oristano | 60 to 69 | 47 | 50 (41 to 61) | -3 (-14 to 6) | 0.93 (0.77 to 1.14) |
| Insular | Sardegna | Oristano | 70 to 79 | 106 | 92 (76 to 111) | 14 (-5 to 30) | 1.15 (0.95 to 1.40) |
| Insular | Sardegna | Oristano | 80 to 89 | 204 | 191 (149 to 230) | 13 (-26 to 55) | 1.07 (0.89 to 1.37) |

| Macroregion | Region | Province | Age group | Observed deaths | Expected baseline | Excess deaths | SMRs |
|---|---|---|---|---|---|---|---|
| Insular | Sardegna | Oristano | 90+ | 134 | 120 (95 to 145) | 14 (-11 to 39) | 1.12 (0.93 to 1.42) |
| Insular | Sardegna | Sassari | 0 to 59 | 101 | 108 (97 to 119) | -7 (-18 to 4) | 0.94 (0.85 to 1.04) |
| Insular | Sardegna | Sassari | 60 to 69 | 133 | 186 (155 to 219) | -53 (-86 to -22) | 0.71 (0.61 to 0.86) |
| Insular | Sardegna | Sassari | 70 to 79 | 255 | 352 (289 to 416) | -97 (-161 to -34) | 0.72 (0.61 to 0.88) |
| Insular | Sardegna | Sassari | 80 to 89 | 537 | 599 (477 to 725) | -62 (-188 to 60) | 0.90 (0.74 to 1.13) |
| Insular | Sardegna | Sassari | 90+ | 317 | 287 (226 to 346) | 30 (-29 to 91) | 1.10 (0.92 to 1.40) |
| Insular | Sardegna | Sud Sardegna | 0 to 59 | 89 | 75 (67 to 83) | 14 (6 to 22) | 1.19 (1.07 to 1.33) |
| Insular | Sardegna | Sud Sardegna | 60 to 69 | 104 | 104 (85 to 122) | 0 (-18 to 19) | 1.00 (0.85 to 1.22) |
| Insular | Sardegna | Sud Sardegna | 70 to 79 | 218 | 182 (151 to 216) | 36 (2 to 67) | 1.20 (1.01 to 1.44) |
| Insular | Sardegna | Sud Sardegna | 80 to 89 | 376 | 352 (277 to 422) | 24 (-46 to 99) | 1.07 (0.89 to 1.36) |
| Insular | Sardegna | Sud Sardegna | 90+ | 283 | 243 (193 to 295) | 40 (-12 to 90) | 1.17 (0.96 to 1.47) |
| Insular | Sicilia | Agrigento | 0 to 59 | 100 | 86 (78 to 95) | 14 (5 to 22) | 1.16 (1.06 to 1.28) |
| Insular | Sicilia | Agrigento | 60 to 69 | 98 | 112 (93 to 133) | -14 (-35 to 5) | 0.88 (0.74 to 1.05) |
| Insular | Sicilia | Agrigento | 70 to 79 | 262 | 247 (202 to 291) | 15 (-29 to 60) | 1.06 (0.90 to 1.30) |
| Insular | Sicilia | Agrigento | 80 to 89 | 522 | 531 (420 to 637) | -9 (-115 to 102) | 0.98 (0.82 to 1.24) |
| Insular | Sicilia | Agrigento | 90+ | 292 | 284 (227 to 341) | 8 (-49 to 65) | 1.03 (0.86 to 1.29) |
| Insular | Sicilia | Caltanissetta | 0 to 59 | 52 | 57 (51 to 64) | -5 (-12 to 1) | 0.91 (0.82 to 1.01) |
| Insular | Sicilia | Caltanissetta | 60 to 69 | 85 | 67 (56 to 81) | 18 (4 to 29) | 1.26 (1.05 to 1.52) |
| Insular | Sicilia | Caltanissetta | 70 to 79 | 164 | 155 (129 to 185) | 9 (-21 to 35) | 1.06 (0.89 to 1.27) |
| Insular | Sicilia | Caltanissetta | 80 to 89 | 304 | 312 (246 to 376) | -8 (-72 to 58) | 0.98 (0.81 to 1.24) |
| Insular | Sicilia | Caltanissetta | 90+ | 164 | 148 (117 to 180) | 16 (-16 to 47) | 1.10 (0.91 to 1.40) |
| Insular | Sicilia | Catania | 0 to 59 | 205 | 214 (199 to 231) | -9 (-26 to 6) | 0.96 (0.89 to 1.03) |
| Insular | Sicilia | Catania | 60 to 69 | 260 | 282 (236 to 332) | -22 (-72 to 24) | 0.92 (0.78 to 1.10) |
| Insular | Sicilia | Catania | 70 to 79 | 537 | 560 (462 to 660) | -23 (-123 to 75) | 0.96 (0.81 to 1.16) |
| Insular | Sicilia | Catania | 80 to 89 | 1,054 | 1,082 (854 to 1,302) | -28 (-248 to 200) | 0.97 (0.81 to 1.23) |
| Insular | Sicilia | Catania | 90+ | 583 | 572 (453 to 691) | 11 (-108 to 130) | 1.02 (0.84 to 1.29) |
| Insular | Sicilia | Enna | 0 to 59 | 43 | 34 (30 to 39) | 9 (4 to 13) | 1.25 (1.09 to 1.42) |
| Insular | Sicilia | Enna | 60 to 69 | 53 | 46 (37 to 56) | 7 (-3 to 16) | 1.15 (0.95 to 1.42) |
| Insular | Sicilia | Enna | 70 to 79 | 111 | 96 (78 to 114) | 15 (-3 to 33) | 1.16 (0.97 to 1.42) |
| Insular | Sicilia | Enna | 80 to 89 | 237 | 223 (176 to 269) | 14 (-32 to 61) | 1.06 (0.88 to 1.35) |
| Insular | Sicilia | Enna | 90+ | 128 | 124 (98 to 150) | 4 (-22 to 30) | 1.03 (0.85 to 1.31) |
| Insular | Sicilia | Messina | 0 to 59 | 157 | 115 (105 to 126) | 42 (31 to 52) | 1.37 (1.25 to 1.49) |
| Insular | Sicilia | Messina | 60 to 69 | 191 | 180 (150 to 211) | 11 (-20 to 41) | 1.06 (0.91 to 1.27) |
| Insular | Sicilia | Messina | 70 to 79 | 382 | 363 (301 to 430) | 19 (-48 to 81) | 1.05 (0.89 to 1.27) |
| Insular | Sicilia | Messina | 80 to 89 | 729 | 731 (581 to 883) | -2 (-154 to 148) | 1.00 (0.83 to 1.25) |
| Insular | Sicilia | Messina | 90+ | 489 | 438 (348 to 525) | 51 (-36 to 141) | 1.12 (0.93 to 1.41) |
| Insular | Sicilia | Palermo | 0 to 59 | 223 | 248 (232 to 266) | -25 (-43 to -9) | 0.90 (0.84 to 0.96) |
| Insular | Sicilia | Palermo | 60 to 69 | 336 | 324 (272 to 381) | 12 (-45 to 64) | 1.04 (0.88 to 1.24) |
| Insular | Sicilia | Palermo | 70 to 79 | 714 | 671 (552 to 793) | 43 (-79 to 162) | 1.06 (0.90 to 1.29) |
| Insular | Sicilia | Palermo | 80 to 89 | 1,284 | 1,268 (1,003 to 1,520) | 16 (-236 to 281) | 1.01 (0.84 to 1.28) |
| Insular | Sicilia | Palermo | 90+ | 748 | 711 (568 to 854) | 37 (-106 to 180) | 1.05 (0.88 to 1.32) |
| Insular | Sicilia | Ragusa | 0 to 59 | 57 | 60 (54 to 68) | -3 (-11 to 3) | 0.95 (0.84 to 1.05) |
| Insular | Sicilia | Ragusa | 60 to 69 | 72 | 67 (55 to 81) | 5 (-9 to 17) | 1.07 (0.89 to 1.30) |
| Insular | Sicilia | Ragusa | 70 to 79 | 154 | 153 (126 to 181) | 1 (-27 to 28) | 1.01 (0.85 to 1.22) |

| Macroregion | Region | Province | Age group | Observed deaths | Expected baseline | Excess deaths | SMRs |
|---|---|---|---|---|---|---|---|
| Insular | Sicilia | Ragusa | 80 to 89 | 343 | 329 (259 to 397) | 14 (-54 to 84) | 1.04 (0.86 to 1.32) |
| Insular | Sicilia | Ragusa | 90+ | 206 | 190 (152 to 231) | 16 (-25 to 54) | 1.08 (0.89 to 1.35) |
| Insular | Sicilia | Siracusa | 0 to 59 | 75 | 77 (69 to 85) | -2 (-10 to 6) | 0.98 (0.88 to 1.09) |
| Insular | Sicilia | Siracusa | 60 to 69 | 113 | 100 (83 to 117) | 13 (-4 to 30) | 1.13 (0.96 to 1.36) |
| Insular | Sicilia | Siracusa | 70 to 79 | 269 | 229 (189 to 272) | 40 (-3 to 80) | 1.17 (0.99 to 1.43) |
| Insular | Sicilia | Siracusa | 80 to 89 | 444 | 419 (331 to 502) | 25 (-58 to 113) | 1.06 (0.88 to 1.34) |
| Insular | Sicilia | Siracusa | 90+ | 238 | 196 (157 to 237) | 42 (1 to 81) | 1.21 (1.00 to 1.52) |
| Insular | Sicilia | Trapani | 0 to 59 | 76 | 88 (80 to 97) | -12 (-21 to -4) | 0.86 (0.78 to 0.95) |
| Insular | Sicilia | Trapani | 60 to 69 | 83 | 106 (87 to 125) | -23 (-42 to -4) | 0.78 (0.66 to 0.95) |
| Insular | Sicilia | Trapani | 70 to 79 | 257 | 228 (189 to 271) | 29 (-14 to 68) | 1.13 (0.95 to 1.36) |
| Insular | Sicilia | Trapani | 80 to 89 | 481 | 493 (392 to 595) | -12 (-114 to 89) | 0.98 (0.81 to 1.23) |
| Insular | Sicilia | Trapani | 90+ | 313 | 285 (225 to 342) | 28 (-29 to 88) | 1.10 (0.91 to 1.39) |
| Northeast | Emilia-Romagna | Bologna | 0 to 59 | 191 | 161 (151 to 173) | 30 (18 to 40) | 1.19 (1.10 to 1.26) |
| Northeast | Emilia-Romagna | Bologna | 60 to 69 | 254 | 213 (179 to 250) | 41 (4 to 75) | 1.19 (1.02 to 1.42) |
| Northeast | Emilia-Romagna | Bologna | 70 to 79 | 657 | 480 (393 to 563) | 177 (94 to 264) | 1.37 (1.17 to 1.67) |
| Northeast | Emilia-Romagna | Bologna | 80 to 89 | 1,433 | 1,100 (871 to 1,324) | 333 (109 to 562) | 1.30 (1.08 to 1.65) |
| Northeast | Emilia-Romagna | Bologna | 90+ | 1,135 | 824 (660 to 987) | 311 (148 to 475) | 1.38 (1.15 to 1.72) |
| Northeast | Emilia-Romagna | Ferrara | 0 to 59 | 81 | 65 (60 to 71) | 16 (10 to 21) | 1.24 (1.14 to 1.36) |
| Northeast | Emilia-Romagna | Ferrara | 60 to 69 | 99 | 97 (80 to 115) | 2 (-16 to 19) | 1.02 (0.86 to 1.23) |
| Northeast | Emilia-Romagna | Ferrara | 70 to 79 | 245 | 210 (172 to 249) | 35 (-4 to 73) | 1.17 (0.98 to 1.42) |
| Northeast | Emilia-Romagna | Ferrara | 80 to 89 | 549 | 478 (379 to 576) | 71 (-27 to 170) | 1.15 (0.95 to 1.45) |
| Northeast | Emilia-Romagna | Ferrara | 90+ | 350 | 297 (236 to 357) | 53 (-7 to 114) | 1.18 (0.98 to 1.48) |
| Northeast | Emilia-Romagna | Forli'-Cesena | 0 to 59 | 62 | 65 (59 to 72) | -3 (-10 to 3) | 0.96 (0.87 to 1.06) |
| Northeast | Emilia-Romagna | Forli'-Cesena | 60 to 69 | 113 | 81 (67 to 96) | 32 (17 to 46) | 1.39 (1.17 to 1.68) |
| Northeast | Emilia-Romagna | Forli'-Cesena | 70 to 79 | 247 | 179 (147 to 213) | 68 (34 to 100) | 1.38 (1.16 to 1.68) |
| Northeast | Emilia-Romagna | Forli'-Cesena | 80 to 89 | 550 | 422 (332 to 510) | 128 (40 to 218) | 1.30 (1.08 to 1.65) |
| Northeast | Emilia-Romagna | Forli'-Cesena | 90+ | 389 | 313 (250 to 376) | 76 (13 to 139) | 1.24 (1.03 to 1.55) |
| Northeast | Emilia-Romagna | Modena | 0 to 59 | 136 | 115 (106 to 124) | 21 (12 to 30) | 1.18 (1.09 to 1.28) |
| Northeast | Emilia-Romagna | Modena | 60 to 69 | 173 | 143 (119 to 168) | 30 (5 to 54) | 1.21 (1.03 to 1.45) |
| Northeast | Emilia-Romagna | Modena | 70 to 79 | 437 | 318 (261 to 374) | 119 (63 to 176) | 1.38 (1.17 to 1.67) |
| Northeast | Emilia-Romagna | Modena | 80 to 89 | 922 | 683 (535 to 825) | 239 (97 to 387) | 1.35 (1.12 to 1.72) |
| Northeast | Emilia-Romagna | Modena | 90+ | 672 | 496 (396 to 597) | 176 (75 to 276) | 1.36 (1.13 to 1.70) |
| Northeast | Emilia-Romagna | Parma | 0 to 59 | 115 | 77 (70 to 85) | 38 (30 to 45) | 1.49 (1.36 to 1.63) |
| Northeast | Emilia-Romagna | Parma | 60 to 69 | 227 | 92 (75 to 109) | 135 (118 to 152) | 2.47 (2.09 to 3.01) |
| Northeast | Emilia-Romagna | Parma | 70 to 79 | 595 | 208 (171 to 250) | 387 (345 to 424) | 2.86 (2.38 to 3.49) |
| Northeast | Emilia-Romagna | Parma | 80 to 89 | 1,071 | 470 (369 to 568) | 601 (503 to 702) | 2.28 (1.89 to 2.90) |
| Northeast | Emilia-Romagna | Parma | 90+ | 692 | 343 (270 to 412) | 349 (280 to 422) | 2.02 (1.68 to 2.56) |
| Northeast | Emilia-Romagna | Piacenza | 0 to 59 | 83 | 49 (44 to 54) | 34 (29 to 39) | 1.71 (1.53 to 1.91) |
| Northeast | Emilia-Romagna | Piacenza | 60 to 69 | 185 | 67 (55 to 80) | 118 (105 to 130) | 2.77 (2.31 to 3.38) |
| Northeast | Emilia-Romagna | Piacenza | 70 to 79 | 504 | 154 (126 to 184) | 350 (320 to 378) | 3.28 (2.74 to 4.00) |
| Northeast | Emilia-Romagna | Piacenza | 80 to 89 | 880 | 347 (274 to 417) | 533 (463 to 606) | 2.54 (2.11 to 3.22) |
| Northeast | Emilia-Romagna | Piacenza | 90+ | 557 | 241 (192 to 289) | 316 (268 to 365) | 2.31 (1.93 to 2.91) |
| Northeast | Emilia-Romagna | Ravenna | 0 to 59 | 61 | 67 (60 to 73) | -6 (-12 to 1) | 0.91 (0.83 to 1.01) |
| Northeast | Emilia-Romagna | Ravenna | 60 to 69 | 83 | 83 (69 to 100) | 0 (-17 to 14) | 1.00 (0.83 to 1.20) |

| Macroregion | Region | Province | Age group | Observed deaths | Expected baseline | Excess deaths | SMRs |
|---|---|---|---|---|---|---|---|
| Northeast | Emilia-Romagna | Ravenna | 70 to 79 | 207 | 183 (150 to 215) | 24 (-8 to 57) | 1.13 (0.96 to 1.38) |
| Northeast | Emilia-Romagna | Ravenna | 80 to 89 | 502 | 442 (350 to 532) | 60 (-30 to 152) | 1.14 (0.94 to 1.43) |
| Northeast | Emilia-Romagna | Ravenna | 90+ | 412 | 344 (275 to 412) | 68 (0 to 137) | 1.20 (1.00 to 1.50) |
| Northeast | Emilia-Romagna | Reggio nell'Emilia | 0 to 59 | 95 | 96 (88 to 104) | -1 (-9 to 7) | 0.99 (0.91 to 1.08) |
| Northeast | Emilia-Romagna | Reggio nell'Emilia | 60 to 69 | 156 | 110 (91 to 130) | 46 (26 to 65) | 1.42 (1.20 to 1.71) |
| Northeast | Emilia-Romagna | Reggio nell'Emilia | 70 to 79 | 380 | 238 (197 to 281) | 142 (99 to 183) | 1.60 (1.35 to 1.93) |
| Northeast | Emilia-Romagna | Reggio nell'Emilia | 80 to 89 | 788 | 512 (406 to 614) | 276 (174 to 382) | 1.54 (1.28 to 1.94) |
| Northeast | Emilia-Romagna | Reggio nell'Emilia | 90+ | 587 | 369 (293 to 444) | 218 (143 to 294) | 1.59 (1.32 to 2.00) |
| Northeast | Emilia-Romagna | Rimini | 0 to 59 | 72 | 54 (48 to 61) | 18 (11 to 24) | 1.33 (1.19 to 1.49) |
| Northeast | Emilia-Romagna | Rimini | 60 to 69 | 90 | 63 (52 to 75) | 27 (15 to 38) | 1.42 (1.20 to 1.74) |
| Northeast | Emilia-Romagna | Rimini | 70 to 79 | 212 | 140 (114 to 166) | 72 (46 to 98) | 1.52 (1.27 to 1.87) |
| Northeast | Emilia-Romagna | Rimini | 80 to 89 | 464 | 320 (253 to 383) | 144 (81 to 211) | 1.45 (1.21 to 1.83) |
| Northeast | Emilia-Romagna | Rimini | 90+ | 337 | 230 (182 to 279) | 107 (58 to 155) | 1.47 (1.21 to 1.85) |
| Northeast | Friuli-Venezia Giulia | Gorizia | 0 to 59 | 27 | 28 (24 to 32) | -1 (-5 to 3) | 0.97 (0.83 to 1.13) |
| Northeast | Friuli-Venezia Giulia | Gorizia | 60 to 69 | 32 | 38 (31 to 46) | -6 (-14 to 1) | 0.84 (0.70 to 1.04) |
| Northeast | Friuli-Venezia Giulia | Gorizia | 70 to 79 | 89 | 88 (71 to 106) | 1 (-17 to 18) | 1.01 (0.84 to 1.25) |
| Northeast | Friuli-Venezia Giulia | Gorizia | 80 to 89 | 190 | 167 (129 to 203) | 23 (-13 to 61) | 1.14 (0.94 to 1.47) |
| Northeast | Friuli-Venezia Giulia | Gorizia | 90+ | 127 | 118 (93 to 143) | 9 (-16 to 34) | 1.07 (0.89 to 1.36) |
| Northeast | Friuli-Venezia Giulia | Pordenone | 0 to 59 | 46 | 51 (46 to 57) | -5 (-11 to 0) | 0.89 (0.80 to 1.00) |
| Northeast | Friuli-Venezia Giulia | Pordenone | 60 to 69 | 82 | 65 (54 to 78) | 17 (4 to 28) | 1.27 (1.06 to 1.53) |
| Northeast | Friuli-Venezia Giulia | Pordenone | 70 to 79 | 180 | 155 (129 to 184) | 25 (-4 to 51) | 1.16 (0.98 to 1.40) |
| Northeast | Friuli-Venezia Giulia | Pordenone | 80 to 89 | 310 | 294 (231 to 356) | 16 (-46 to 79) | 1.05 (0.87 to 1.34) |
| Northeast | Friuli-Venezia Giulia | Pordenone | 90+ | 290 | 226 (180 to 272) | 64 (18 to 110) | 1.28 (1.07 to 1.61) |
| Northeast | Friuli-Venezia Giulia | Trieste | 0 to 59 | 51 | 46 (40 to 52) | 5 (-1 to 11) | 1.11 (0.98 to 1.29) |
| Northeast | Friuli-Venezia Giulia | Trieste | 60 to 69 | 65 | 60 (49 to 72) | 5 (-7 to 16) | 1.08 (0.90 to 1.31) |
| Northeast | Friuli-Venezia Giulia | Trieste | 70 to 79 | 184 | 163 (134 to 195) | 21 (-11 to 50) | 1.13 (0.94 to 1.37) |
| Northeast | Friuli-Venezia Giulia | Trieste | 80 to 89 | 393 | 302 (237 to 363) | 91 (30 to 156) | 1.30 (1.08 to 1.66) |
| Northeast | Friuli-Venezia Giulia | Trieste | 90+ | 301 | 229 (184 to 274) | 72 (27 to 117) | 1.32 (1.10 to 1.64) |
| Northeast | Friuli-Venezia Giulia | Udine | 0 to 59 | 87 | 92 (83 to 100) | -5 (-13 to 4) | 0.95 (0.87 to 1.04) |
| Northeast | Friuli-Venezia Giulia | Udine | 60 to 69 | 149 | 142 (119 to 168) | 7 (-19 to 30) | 1.05 (0.88 to 1.25) |
| Northeast | Friuli-Venezia Giulia | Udine | 70 to 79 | 361 | 321 (265 to 379) | 40 (-18 to 96) | 1.12 (0.95 to 1.36) |
| Northeast | Friuli-Venezia Giulia | Udine | 80 to 89 | 618 | 603 (475 to 727) | 15 (-109 to 143) | 1.02 (0.85 to 1.30) |
| Northeast | Friuli-Venezia Giulia | Udine | 90+ | 460 | 432 (344 to 518) | 28 (-58 to 116) | 1.07 (0.89 to 1.34) |
| Northeast | Trentino-Alto Adige | Bolzano | 0 to 59 | 100 | 86 (79 to 95) | 14 (5 to 21) | 1.16 (1.06 to 1.27) |

| Macroregion | Region | Province | Age group | Observed deaths | Expected baseline | Excess deaths | SMRs |
|---|---|---|---|---|---|---|---|
| Northeast | Trentino-Alto Adige | Bolzano | 60 to 69 | 143 | 95 (79 to 113) | 48 (30 to 64) | 1.50 (1.26 to 1.80) |
| Northeast | Trentino-Alto Adige | Bolzano | 70 to 79 | 304 | 205 (169 to 242) | 99 (62 to 135) | 1.48 (1.25 to 1.79) |
| Northeast | Trentino-Alto Adige | Bolzano | 80 to 89 | 616 | 411 (326 to 495) | 205 (121 to 290) | 1.50 (1.24 to 1.89) |
| Northeast | Trentino-Alto Adige | Bolzano | 90+ | 445 | 300 (240 to 363) | 145 (82 to 205) | 1.48 (1.23 to 1.86) |
| Northeast | Trentino-Alto Adige | Trento | 0 to 59 | 100 | 87 (79 to 94) | 13 (6 to 21) | 1.15 (1.06 to 1.26) |
| Northeast | Trentino-Alto Adige | Trento | 60 to 69 | 116 | 110 (93 to 130) | 6 (-14 to 23) | 1.05 (0.89 to 1.25) |
| Northeast | Trentino-Alto Adige | Trento | 70 to 79 | 352 | 231 (189 to 271) | 121 (81 to 163) | 1.53 (1.30 to 1.86) |
| Northeast | Trentino-Alto Adige | Trento | 80 to 89 | 768 | 473 (375 to 569) | 295 (199 to 393) | 1.63 (1.35 to 2.05) |
| Northeast | Trentino-Alto Adige | Trento | 90+ | 637 | 364 (290 to 442) | 273 (195 to 347) | 1.75 (1.44 to 2.20) |
| Northeast | Veneto | Belluno | 0 to 59 | 26 | 36 (32 to 40) | -10 (-14 to -6) | 0.73 (0.64 to 0.82) |
| Northeast | Veneto | Belluno | 60 to 69 | 58 | 58 (48 to 70) | 0 (-12 to 10) | 1.00 (0.83 to 1.21) |
| Northeast | Veneto | Belluno | 70 to 79 | 145 | 123 (100 to 146) | 22 (-1 to 45) | 1.18 (0.99 to 1.45) |
| Northeast | Veneto | Belluno | 80 to 89 | 277 | 236 (186 to 284) | 41 (-7 to 91) | 1.18 (0.97 to 1.49) |
| Northeast | Veneto | Belluno | 90+ | 220 | 175 (138 to 211) | 45 (9 to 82) | 1.26 (1.04 to 1.59) |
| Northeast | Veneto | Padova | 0 to 59 | 143 | 155 (145 to 166) | -12 (-23 to -2) | 0.92 (0.86 to 0.99) |
| Northeast | Veneto | Padova | 60 to 69 | 188 | 194 (161 to 230) | -6 (-42 to 27) | 0.97 (0.82 to 1.16) |
| Northeast | Veneto | Padova | 70 to 79 | 465 | 414 (344 to 488) | 51 (-23 to 121) | 1.12 (0.95 to 1.35) |
| Northeast | Veneto | Padova | 80 to 89 | 970 | 876 (693 to 1,056) | 94 (-86 to 277) | 1.11 (0.92 to 1.40) |
| Northeast | Veneto | Padova | 90+ | 750 | 607 (485 to 731) | 143 (19 to 265) | 1.24 (1.03 to 1.55) |
| Northeast | Veneto | Rovigo | 0 to 59 | 61 | 42 (38 to 48) | 19 (13 to 23) | 1.44 (1.28 to 1.61) |
| Northeast | Veneto | Rovigo | 60 to 69 | 78 | 66 (54 to 78) | 12 (0 to 24) | 1.19 (1.00 to 1.43) |
| Northeast | Veneto | Rovigo | 70 to 79 | 142 | 141 (116 to 168) | 1 (-26 to 26) | 1.00 (0.84 to 1.22) |
| Northeast | Veneto | Rovigo | 80 to 89 | 340 | 298 (234 to 359) | 42 (-19 to 106) | 1.14 (0.95 to 1.46) |
| Northeast | Veneto | Rovigo | 90+ | 250 | 196 (154 to 237) | 54 (13 to 96) | 1.28 (1.06 to 1.62) |
| Northeast | Veneto | Treviso | 0 to 59 | 133 | 139 (129 to 150) | -6 (-17 to 4) | 0.96 (0.89 to 1.03) |
| Northeast | Veneto | Treviso | 60 to 69 | 185 | 182 (151 to 213) | 3 (-28 to 34) | 1.02 (0.87 to 1.23) |
| Northeast | Veneto | Treviso | 70 to 79 | 440 | 377 (311 to 446) | 63 (-6 to 129) | 1.17 (0.99 to 1.42) |
| Northeast | Veneto | Treviso | 80 to 89 | 961 | 776 (611 to 937) | 185 (24 to 350) | 1.24 (1.03 to 1.57) |
| Northeast | Veneto | Treviso | 90+ | 786 | 586 (468 to 705) | 200 (81 to 318) | 1.34 (1.11 to 1.68) |
| Northeast | Veneto | Venezia | 0 to 59 | 158 | 135 (125 to 145) | 23 (13 to 33) | 1.17 (1.09 to 1.26) |
| Northeast | Veneto | Venezia | 60 to 69 | 203 | 199 (168 to 233) | 4 (-30 to 35) | 1.02 (0.87 to 1.21) |
| Northeast | Veneto | Venezia | 70 to 79 | 555 | 451 (373 to 533) | 104 (22 to 182) | 1.23 (1.04 to 1.49) |
| Northeast | Veneto | Venezia | 80 to 89 | 1,050 | 891 (698 to 1,077) | 159 (-27 to 352) | 1.18 (0.98 to 1.50) |
| Northeast | Veneto | Venezia | 90+ | 746 | 566 (449 to 680) | 180 (66 to 297) | 1.32 (1.10 to 1.66) |
| Northeast | Veneto | Verona | 0 to 59 | 174 | 147 (137 to 158) | 27 (16 to 37) | 1.18 (1.10 to 1.27) |
| Northeast | Veneto | Verona | 60 to 69 | 199 | 185 (155 to 217) | 14 (-18 to 44) | 1.08 (0.92 to 1.29) |
| Northeast | Veneto | Verona | 70 to 79 | 517 | 418 (347 to 494) | 99 (23 to 170) | 1.24 (1.05 to 1.49) |
| Northeast | Veneto | Verona | 80 to 89 | 1,151 | 849 (670 to 1,023) | 302 (128 to 481) | 1.35 (1.13 to 1.72) |
| Northeast | Veneto | Verona | 90+ | 852 | 600 (478 to 718) | 252 (134 to 374) | 1.42 (1.19 to 1.78) |
| Northeast | Veneto | Vicenza | 0 to 59 | 133 | 149 (139 to 160) | -16 (-27 to -6) | 0.89 (0.83 to 0.96) |

| Macroregion | Region | Province | Age group | Observed deaths | Expected baseline | Excess deaths | SMRs |
|---|---|---|---|---|---|---|---|
| Northeast | Veneto | Vicenza | 60 to 69 | 185 | 180 (151 to 213) | 5 (-28 to 34) | 1.03 (0.87 to 1.22) |
| Northeast | Veneto | Vicenza | 70 to 79 | 459 | 392 (325 to 465) | 67 (-6 to 134) | 1.17 (0.99 to 1.41) |
| Northeast | Veneto | Vicenza | 80 to 89 | 956 | 782 (620 to 942) | 174 (14 to 336) | 1.22 (1.01 to 1.54) |
| Northeast | Veneto | Vicenza | 90+ | 671 | 517 (411 to 621) | 154 (50 to 260) | 1.30 (1.08 to 1.63) |
| Northwest | Liguria | Genova | 0 to 59 | 195 | 143 (133 to 154) | 52 (41 to 62) | 1.36 (1.27 to 1.46) |
| Northwest | Liguria | Genova | 60 to 69 | 302 | 216 (181 to 253) | 86 (49 to 121) | 1.40 (1.19 to 1.67) |
| Northwest | Liguria | Genova | 70 to 79 | 804 | 512 (420 to 605) | 292 (199 to 384) | 1.57 (1.33 to 1.91) |
| Northwest | Liguria | Genova | 80 to 89 | 1,756 | 1,139 (900 to 1,370) | 617 (386 to 856) | 1.54 (1.28 to 1.95) |
| Northwest | Liguria | Genova | 90+ | 1,297 | 830 (658 to 994) | 467 (303 to 639) | 1.56 (1.30 to 1.97) |
| Northwest | Liguria | Imperia | 0 to 59 | 42 | 39 (34 to 45) | 3 (-3 to 8) | 1.06 (0.94 to 1.22) |
| Northwest | Liguria | Imperia | 60 to 69 | 70 | 57 (47 to 68) | 13 (2 to 23) | 1.22 (1.02 to 1.49) |
| Northwest | Liguria | Imperia | 70 to 79 | 217 | 135 (111 to 160) | 82 (57 to 106) | 1.61 (1.36 to 1.96) |
| Northwest | Liguria | Imperia | 80 to 89 | 447 | 298 (234 to 360) | 149 (87 to 213) | 1.50 (1.24 to 1.91) |
| Northwest | Liguria | Imperia | 90+ | 272 | 195 (155 to 236) | 77 (36 to 117) | 1.39 (1.15 to 1.75) |
| Northwest | Liguria | La Spezia | 0 to 59 | 49 | 40 (35 to 45) | 9 (4 to 14) | 1.22 (1.09 to 1.38) |
| Northwest | Liguria | La Spezia | 60 to 69 | 77 | 51 (42 to 61) | 26 (16 to 35) | 1.51 (1.26 to 1.84) |
| Northwest | Liguria | La Spezia | 70 to 79 | 206 | 127 (104 to 150) | 79 (56 to 102) | 1.63 (1.37 to 1.99) |
| Northwest | Liguria | La Spezia | 80 to 89 | 389 | 279 (220 to 337) | 110 (52 to 169) | 1.39 (1.15 to 1.77) |
| Northwest | Liguria | La Spezia | 90+ | 267 | 211 (167 to 256) | 56 (11 to 100) | 1.27 (1.04 to 1.60) |
| Northwest | Liguria | Savona | 0 to 59 | 54 | 45 (40 to 51) | 9 (3 to 14) | 1.19 (1.06 to 1.34) |
| Northwest | Liguria | Savona | 60 to 69 | 92 | 69 (57 to 83) | 23 (9 to 35) | 1.33 (1.11 to 1.61) |
| Northwest | Liguria | Savona | 70 to 79 | 241 | 175 (145 to 208) | 66 (33 to 96) | 1.38 (1.16 to 1.67) |
| Northwest | Liguria | Savona | 80 to 89 | 521 | 396 (314 to 477) | 125 (44 to 207) | 1.32 (1.09 to 1.66) |
| Northwest | Liguria | Savona | 90+ | 375 | 266 (213 to 319) | 109 (56 to 162) | 1.41 (1.17 to 1.76) |
| Northwest | Lombardia | Bergamo | 0 to 59 | 410 | 183 (172 to 196) | 227 (214 to 238) | 2.24 (2.10 to 2.39) |
| Northwest | Lombardia | Bergamo | 60 to 69 | 841 | 247 (206 to 290) | 594 (551 to 635) | 3.40 (2.90 to 4.08) |
| Northwest | Lombardia | Bergamo | 70 to 79 | 2,154 | 521 (431 to 612) | 1,633 (1,542 to 1,723) | 4.13 (3.52 to 4.99) |
| Northwest | Lombardia | Bergamo | 80 to 89 | 3,505 | 952 (756 to 1,149) | 2,553 (2,356 to 2,749) | 3.68 (3.05 to 4.64) |
| Northwest | Lombardia | Bergamo | 90+ | 1,792 | 549 (436 to 661) | 1,243 (1,131 to 1,356) | 3.26 (2.71 to 4.11) |
| Northwest | Lombardia | Brescia | 0 to 59 | 336 | 196 (184 to 209) | 140 (127 to 152) | 1.71 (1.61 to 1.83) |
| Northwest | Lombardia | Brescia | 60 to 69 | 627 | 261 (220 to 306) | 366 (321 to 407) | 2.40 (2.05 to 2.85) |
| Northwest | Lombardia | Brescia | 70 to 79 | 1,719 | 563 (465 to 661) | 1,156 (1,058 to 1,254) | 3.06 (2.60 to 3.70) |
| Northwest | Lombardia | Brescia | 80 to 89 | 2,957 | 1,116 (879 to 1,345) | 1,841 (1,612 to 2,078) | 2.65 (2.20 to 3.37) |
| Northwest | Lombardia | Brescia | 90+ | 1,835 | 749 (597 to 892) | 1,086 (943 to 1,238) | 2.45 (2.06 to 3.07) |
| Northwest | Lombardia | Como | 0 to 59 | 144 | 97 (88 to 105) | 47 (39 to 56) | 1.48 (1.37 to 1.63) |
| Northwest | Lombardia | Como | 60 to 69 | 180 | 137 (116 to 162) | 43 (18 to 64) | 1.31 (1.11 to 1.56) |
| Northwest | Lombardia | Como | 70 to 79 | 469 | 294 (243 to 349) | 175 (120 to 226) | 1.59 (1.34 to 1.93) |
| Northwest | Lombardia | Como | 80 to 89 | 951 | 587 (468 to 707) | 364 (244 to 483) | 1.62 (1.35 to 2.03) |
| Northwest | Lombardia | Como | 90+ | 596 | 383 (304 to 461) | 213 (135 to 292) | 1.56 (1.29 to 1.96) |
| Northwest | Lombardia | Cremona | 0 to 59 | 132 | 59 (53 to 64) | 73 (68 to 79) | 2.25 (2.05 to 2.48) |
| Northwest | Lombardia | Cremona | 60 to 69 | 226 | 82 (68 to 98) | 144 (128 to 158) | 2.75 (2.31 to 3.30) |
| Northwest | Lombardia | Cremona | 70 to 79 | 713 | 196 (161 to 230) | 517 (483 to 552) | 3.64 (3.10 to 4.43) |
| Northwest | Lombardia | Cremona | 80 to 89 | 1,265 | 402 (320 to 488) | 863 (777 to 945) | 3.14 (2.59 to 3.95) |
| Northwest | Lombardia | Cremona | 90+ | 820 | 249 (197 to 299) | 571 (521 to 623) | 3.30 (2.74 to 4.15) |

| Macroregion | Region | Province | Age group | Observed deaths | Expected baseline | Excess deaths | SMRs |
|---|---|---|---|---|---|---|---|
| Northwest | Lombardia | Lecco | 0 to 59 | 78 | 50 (45 to 56) | 28 (22 to 33) | 1.55 (1.39 to 1.74) |
| Northwest | Lombardia | Lecco | 60 to 69 | 114 | 69 (57 to 81) | 45 (33 to 57) | 1.66 (1.40 to 2.01) |
| Northwest | Lombardia | Lecco | 70 to 79 | 369 | 149 (122 to 177) | 220 (192 to 247) | 2.48 (2.09 to 3.02) |
| Northwest | Lombardia | Lecco | 80 to 89 | 708 | 327 (260 to 395) | 381 (313 to 448) | 2.16 (1.79 to 2.73) |
| Northwest | Lombardia | Lecco | 90+ | 453 | 200 (159 to 240) | 253 (213 to 294) | 2.26 (1.88 to 2.85) |
| Northwest | Lombardia | Lodi | 0 to 59 | 77 | 40 (36 to 46) | 37 (31 to 41) | 1.90 (1.69 to 2.15) |
| Northwest | Lombardia | Lodi | 60 to 69 | 149 | 52 (43 to 63) | 97 (86 to 106) | 2.84 (2.38 to 3.46) |
| Northwest | Lombardia | Lodi | 70 to 79 | 417 | 118 (98 to 140) | 299 (277 to 319) | 3.52 (2.98 to 4.27) |
| Northwest | Lombardia | Lodi | 80 to 89 | 578 | 221 (175 to 266) | 357 (312 to 403) | 2.62 (2.17 to 3.30) |
| Northwest | Lombardia | Lodi | 90+ | 317 | 127 (100 to 154) | 190 (163 to 217) | 2.50 (2.05 to 3.18) |
| Northwest | Lombardia | Mantova | 0 to 59 | 96 | 74 (67 to 81) | 22 (15 to 29) | 1.29 (1.19 to 1.42) |
| Northwest | Lombardia | Mantova | 60 to 69 | 138 | 87 (72 to 103) | 51 (35 to 66) | 1.59 (1.34 to 1.91) |
| Northwest | Lombardia | Mantova | 70 to 79 | 384 | 197 (162 to 231) | 187 (153 to 222) | 1.95 (1.66 to 2.37) |
| Northwest | Lombardia | Mantova | 80 to 89 | 878 | 428 (339 to 514) | 450 (364 to 539) | 2.05 (1.71 to 2.59) |
| Northwest | Lombardia | Mantova | 90+ | 569 | 314 (248 to 377) | 255 (192 to 321) | 1.81 (1.51 to 2.30) |
| Northwest | Lombardia | Milano | 0 to 59 | 654 | 531 (506 to 556) | 123 (98 to 148) | 1.23 (1.18 to 1.29) |
| Northwest | Lombardia | Milano | 60 to 69 | 982 | 637 (539 to 742) | 345 (240 to 443) | 1.54 (1.32 to 1.82) |
| Northwest | Lombardia | Milano | 70 to 79 | 2,684 | 1,491 (1,230 to 1,754) | 1,193 (930 to 1,454) | 1.80 (1.53 to 2.18) |
| Northwest | Lombardia | Milano | 80 to 89 | 5,684 | 3,034 (2,399 to 3,640) | 2,650 (2,044 to 3,285) | 1.87 (1.56 to 2.37) |
| Northwest | Lombardia | Milano | 90+ | 3,745 | 1,864 (1,488 to 2,230) | 1,881 (1,515 to 2,257) | 2.01 (1.68 to 2.52) |
| Northwest | Lombardia | Monza e della Brianza | 0 to 59 | 176 | 129 (120 to 139) | 47 (37 to 56) | 1.37 (1.27 to 1.47) |
| Northwest | Lombardia | Monza e della Brianza | 60 to 69 | 267 | 173 (145 to 203) | 94 (64 to 122) | 1.54 (1.31 to 1.84) |
| Northwest | Lombardia | Monza e della Brianza | 70 to 79 | 680 | 379 (314 to 449) | 301 (231 to 366) | 1.79 (1.51 to 2.17) |
| Northwest | Lombardia | Monza e della Brianza | 80 to 89 | 1,401 | 789 (624 to 950) | 612 (451 to 777) | 1.78 (1.47 to 2.24) |
| Northwest | Lombardia | Monza e della Brianza | 90+ | 807 | 449 (359 to 541) | 358 (266 to 448) | 1.80 (1.49 to 2.25) |
| Northwest | Lombardia | Pavia | 0 to 59 | 155 | 107 (99 to 116) | 48 (39 to 56) | 1.44 (1.34 to 1.56) |
| Northwest | Lombardia | Pavia | 60 to 69 | 319 | 143 (121 to 168) | 176 (151 to 198) | 2.23 (1.90 to 2.64) |
| Northwest | Lombardia | Pavia | 70 to 79 | 695 | 304 (251 to 359) | 391 (336 to 444) | 2.28 (1.93 to 2.77) |
| Northwest | Lombardia | Pavia | 80 to 89 | 1,391 | 652 (512 to 783) | 739 (608 to 879) | 2.13 (1.78 to 2.71) |
| Northwest | Lombardia | Pavia | 90+ | 866 | 429 (340 to 513) | 437 (353 to 526) | 2.02 (1.69 to 2.54) |
| Northwest | Lombardia | Sondrio | 0 to 59 | 56 | 32 (28 to 36) | 24 (20 to 28) | 1.77 (1.57 to 2.01) |
| Northwest | Lombardia | Sondrio | 60 to 69 | 81 | 45 (37 to 55) | 36 (26 to 44) | 1.80 (1.48 to 2.18) |
| Northwest | Lombardia | Sondrio | 70 to 79 | 166 | 97 (80 to 117) | 69 (49 to 86) | 1.70 (1.41 to 2.09) |
| Northwest | Lombardia | Sondrio | 80 to 89 | 342 | 191 (150 to 232) | 151 (110 to 192) | 1.79 (1.48 to 2.27) |
| Northwest | Lombardia | Sondrio | 90+ | 189 | 131 (102 to 159) | 58 (30 to 87) | 1.45 (1.19 to 1.86) |
| Northwest | Lombardia | Varese | 0 to 59 | 168 | 145 (135 to 156) | 23 (12 to 33) | 1.16 (1.07 to 1.25) |
| Northwest | Lombardia | Varese | 60 to 69 | 259 | 196 (163 to 228) | 63 (31 to 96) | 1.32 (1.13 to 1.58) |
| Northwest | Lombardia | Varese | 70 to 79 | 609 | 413 (339 to 486) | 196 (123 to 270) | 1.47 (1.25 to 1.79) |
| Northwest | Lombardia | Varese | 80 to 89 | 1,266 | 913 (718 to 1,099) | 353 (167 to 548) | 1.39 (1.15 to 1.76) |
| Northwest | Lombardia | Varese | 90+ | 885 | 569 (454 to 678) | 316 (207 to 431) | 1.55 (1.31 to 1.95) |
| Northwest | Piemonte | Alessandria | 0 to 59 | 108 | 78 (71 to 85) | 30 (23 to 37) | 1.39 (1.27 to 1.52) |

| Macroregion | Region | Province | Age group | Observed deaths | Expected baseline | Excess deaths | SMRs |
|---|---|---|---|---|---|---|---|
| Northwest | Piemonte | Alessandria | 60 to 69 | 205 | 125 (105 to 148) | 80 (57 to 100) | 1.64 (1.38 to 1.96) |
| Northwest | Piemonte | Alessandria | 70 to 79 | 505 | 265 (218 to 313) | 240 (192 to 287) | 1.91 (1.62 to 2.32) |
| Northwest | Piemonte | Alessandria | 80 to 89 | 1,015 | 576 (457 to 692) | 439 (323 to 558) | 1.76 (1.47 to 2.22) |
| Northwest | Piemonte | Alessandria | 90+ | 678 | 396 (314 to 475) | 282 (203 to 364) | 1.71 (1.43 to 2.16) |
| Northwest | Piemonte | Asti | 0 to 59 | 43 | 39 (34 to 44) | 4 (-1 to 9) | 1.11 (0.99 to 1.25) |
| Northwest | Piemonte | Asti | 60 to 69 | 77 | 55 (46 to 66) | 22 (11 to 31) | 1.39 (1.17 to 1.69) |
| Northwest | Piemonte | Asti | 70 to 79 | 191 | 123 (101 to 146) | 68 (45 to 90) | 1.56 (1.31 to 1.89) |
| Northwest | Piemonte | Asti | 80 to 89 | 387 | 271 (213 to 326) | 116 (61 to 174) | 1.43 (1.19 to 1.82) |
| Northwest | Piemonte | Asti | 90+ | 334 | 204 (162 to 246) | 130 (88 to 172) | 1.64 (1.36 to 2.06) |
| Northwest | Piemonte | Biella | 0 to 59 | 32 | 30 (26 to 35) | 2 (-3 to 6) | 1.05 (0.92 to 1.21) |
| Northwest | Piemonte | Biella | 60 to 69 | 55 | 56 (46 to 67) | -1 (-12 to 9) | 0.98 (0.82 to 1.19) |
| Northwest | Piemonte | Biella | 70 to 79 | 179 | 110 (91 to 131) | 69 (48 to 88) | 1.62 (1.37 to 1.97) |
| Northwest | Piemonte | Biella | 80 to 89 | 423 | 226 (178 to 275) | 197 (148 to 245) | 1.87 (1.54 to 2.37) |
| Northwest | Piemonte | Biella | 90+ | 298 | 163 (129 to 198) | 135 (100 to 169) | 1.83 (1.51 to 2.31) |
| Northwest | Piemonte | Cuneo | 0 to 59 | 116 | 104 (95 to 113) | 12 (3 to 21) | 1.11 (1.03 to 1.22) |
| Northwest | Piemonte | Cuneo | 60 to 69 | 166 | 135 (112 to 161) | 31 (5 to 54) | 1.23 (1.03 to 1.48) |
| Northwest | Piemonte | Cuneo | 70 to 79 | 397 | 315 (261 to 369) | 82 (28 to 136) | 1.26 (1.08 to 1.52) |
| Northwest | Piemonte | Cuneo | 80 to 89 | 904 | 708 (559 to 855) | 196 (49 to 345) | 1.28 (1.06 to 1.62) |
| Northwest | Piemonte | Cuneo | 90+ | 629 | 442 (353 to 529) | 187 (100 to 276) | 1.42 (1.19 to 1.78) |
| Northwest | Piemonte | Novara | 0 to 59 | 92 | 64 (58 to 70) | 28 (22 to 34) | 1.44 (1.31 to 1.60) |
| Northwest | Piemonte | Novara | 60 to 69 | 126 | 93 (77 to 111) | 33 (15 to 49) | 1.35 (1.14 to 1.63) |
| Northwest | Piemonte | Novara | 70 to 79 | 288 | 181 (149 to 214) | 107 (74 to 139) | 1.59 (1.34 to 1.94) |
| Northwest | Piemonte | Novara | 80 to 89 | 671 | 406 (319 to 490) | 265 (181 to 352) | 1.65 (1.37 to 2.10) |
| Northwest | Piemonte | Novara | 90+ | 454 | 259 (206 to 313) | 195 (141 to 248) | 1.75 (1.45 to 2.20) |
| Northwest | Piemonte | Torino | 0 to 59 | 458 | 382 (362 to 402) | 76 (56 to 96) | 1.20 (1.14 to 1.26) |
| Northwest | Piemonte | Torino | 60 to 69 | 716 | 525 (444 to 615) | 191 (101 to 272) | 1.36 (1.17 to 1.61) |
| Northwest | Piemonte | Torino | 70 to 79 | 1,844 | 1,214 (1,001 to 1,437) | 630 (407 to 843) | 1.52 (1.28 to 1.84) |
| Northwest | Piemonte | Torino | 80 to 89 | 3,922 | 2,593 (2,047 to 3,128) | 1,329 (794 to 1,875) | 1.51 (1.25 to 1.92) |
| Northwest | Piemonte | Torino | 90+ | 2,345 | 1,507 (1,203 to 1,802) | 838 (543 to 1,142) | 1.56 (1.30 to 1.95) |
| Northwest | Piemonte | Verbano-Cusio-Ossola | 0 to 59 | 37 | 29 (25 to 33) | 8 (4 to 12) | 1.29 (1.12 to 1.47) |
| Northwest | Piemonte | Verbano-Cusio-Ossola | 60 to 69 | 49 | 45 (37 to 54) | 4 (-5 to 12) | 1.10 (0.91 to 1.34) |
| Northwest | Piemonte | Verbano-Cusio-Ossola | 70 to 79 | 141 | 99 (80 to 117) | 42 (24 to 61) | 1.43 (1.20 to 1.77) |
| Northwest | Piemonte | Verbano-Cusio-Ossola | 80 to 89 | 296 | 196 (153 to 236) | 100 (60 to 143) | 1.51 (1.26 to 1.93) |
| Northwest | Piemonte | Verbano-Cusio-Ossola | 90+ | 167 | 131 (104 to 160) | 36 (7 to 63) | 1.27 (1.04 to 1.61) |
| Northwest | Piemonte | Vercelli | 0 to 59 | 35 | 34 (31 to 39) | 1 (-4 to 4) | 1.02 (0.91 to 1.14) |
| Northwest | Piemonte | Vercelli | 60 to 69 | 84 | 54 (44 to 64) | 30 (20 to 40) | 1.56 (1.30 to 1.89) |
| Northwest | Piemonte | Vercelli | 70 to 79 | 183 | 104 (85 to 123) | 79 (60 to 98) | 1.76 (1.49 to 2.14) |
| Northwest | Piemonte | Vercelli | 80 to 89 | 397 | 232 (181 to 281) | 165 (116 to 216) | 1.71 (1.41 to 2.19) |
| Northwest | Piemonte | Vercelli | 90+ | 250 | 158 (124 to 190) | 92 (60 to 126) | 1.59 (1.31 to 2.02) |
| Northwest | Valle d'Aosta | Aosta | 0 to 59 | 20 | 22 (19 to 26) | -2 (-6 to 1) | 0.90 (0.78 to 1.04) |
| Northwest | Valle d'Aosta | Aosta | 60 to 69 | 50 | 34 (28 to 41) | 16 (9 to 22) | 1.47 (1.21 to 1.79) |

| Macroregion | Region | Province | Age group | Observed deaths | Expected baseline | Excess deaths | SMRs |
|---|---|---|---|---|---|---|---|
| Northwest | Valle d'Aosta | Aosta | 70 to 79 | 104 | 73 (60 to 88) | 31 (16 to 44) | 1.42 (1.18 to 1.73) |
| Northwest | Valle d'Aosta | Aosta | 80 to 89 | 200 | 137 (108 to 167) | 63 (33 to 92) | 1.46 (1.20 to 1.86) |
| Northwest | Valle d'Aosta | Aosta | 90+ | 145 | 97 (76 to 118) | 48 (27 to 69) | 1.50 (1.23 to 1.92) |
| South | Abruzzo | Chieti | 0 to 59 | 84 | 69 (63 to 76) | 15 (8 to 21) | 1.21 (1.10 to 1.34) |
| South | Abruzzo | Chieti | 60 to 69 | 113 | 96 (80 to 113) | 17 (0 to 33) | 1.18 (1.00 to 1.42) |
| South | Abruzzo | Chieti | 70 to 79 | 208 | 207 (171 to 247) | 1 (-39 to 37) | 1.00 (0.84 to 1.21) |
| South | Abruzzo | Chieti | 80 to 89 | 539 | 471 (371 to 568) | 68 (-29 to 168) | 1.14 (0.95 to 1.45) |
| South | Abruzzo | Chieti | 90+ | 341 | 299 (238 to 359) | 42 (-18 to 103) | 1.14 (0.95 to 1.44) |
| South | Abruzzo | L'Aquila | 0 to 59 | 38 | 52 (47 to 58) | -14 (-20 to -9) | 0.73 (0.65 to 0.81) |
| South | Abruzzo | L'Aquila | 60 to 69 | 72 | 76 (62 to 90) | -4 (-18 to 10) | 0.95 (0.80 to 1.15) |
| South | Abruzzo | L'Aquila | 70 to 79 | 157 | 153 (126 to 181) | 4 (-24 to 31) | 1.03 (0.87 to 1.25) |
| South | Abruzzo | L'Aquila | 80 to 89 | 340 | 333 (262 to 403) | 7 (-63 to 78) | 1.02 (0.84 to 1.30) |
| South | Abruzzo | L'Aquila | 90+ | 262 | 243 (191 to 293) | 19 (-31 to 71) | 1.08 (0.90 to 1.37) |
| South | Abruzzo | Pescara | 0 to 59 | 64 | 56 (51 to 62) | 8 (2 to 13) | 1.15 (1.03 to 1.27) |
| South | Abruzzo | Pescara | 60 to 69 | 97 | 65 (54 to 78) | 32 (19 to 43) | 1.48 (1.25 to 1.81) |
| South | Abruzzo | Pescara | 70 to 79 | 209 | 153 (126 to 184) | 56 (25 to 83) | 1.37 (1.14 to 1.66) |
| South | Abruzzo | Pescara | 80 to 89 | 434 | 343 (272 to 415) | 91 (19 to 162) | 1.26 (1.05 to 1.59) |
| South | Abruzzo | Pescara | 90+ | 275 | 220 (173 to 265) | 55 (10 to 102) | 1.25 (1.04 to 1.59) |
| South | Abruzzo | Teramo | 0 to 59 | 59 | 55 (50 to 61) | 4 (-2 to 9) | 1.07 (0.97 to 1.19) |
| South | Abruzzo | Teramo | 60 to 69 | 87 | 73 (60 to 88) | 14 (-1 to 27) | 1.19 (0.99 to 1.46) |
| South | Abruzzo | Teramo | 70 to 79 | 174 | 155 (127 to 185) | 19 (-11 to 47) | 1.12 (0.94 to 1.37) |
| South | Abruzzo | Teramo | 80 to 89 | 396 | 345 (272 to 417) | 51 (-21 to 124) | 1.15 (0.95 to 1.45) |
| South | Abruzzo | Teramo | 90+ | 246 | 198 (157 to 241) | 48 (5 to 89) | 1.24 (1.02 to 1.57) |
| South | Basilicata | Matera | 0 to 59 | 41 | 40 (35 to 45) | 1 (-4 to 6) | 1.02 (0.91 to 1.16) |
| South | Basilicata | Matera | 60 to 69 | 47 | 48 (40 to 58) | -1 (-11 to 7) | 0.98 (0.81 to 1.19) |
| South | Basilicata | Matera | 70 to 79 | 82 | 97 (80 to 116) | -15 (-34 to 2) | 0.85 (0.71 to 1.03) |
| South | Basilicata | Matera | 80 to 89 | 243 | 208 (164 to 250) | 35 (-7 to 79) | 1.17 (0.97 to 1.48) |
| South | Basilicata | Matera | 90+ | 132 | 114 (89 to 137) | 18 (-5 to 43) | 1.16 (0.96 to 1.48) |
| South | Basilicata | Potenza | 0 to 59 | 66 | 72 (66 to 80) | -6 (-14 to 0) | 0.91 (0.83 to 1.01) |
| South | Basilicata | Potenza | 60 to 69 | 103 | 94 (78 to 111) | 9 (-8 to 25) | 1.10 (0.92 to 1.32) |
| South | Basilicata | Potenza | 70 to 79 | 177 | 178 (146 to 210) | -1 (-33 to 31) | 1.00 (0.84 to 1.21) |
| South | Basilicata | Potenza | 80 to 89 | 473 | 453 (356 to 544) | 20 (-71 to 117) | 1.05 (0.87 to 1.33) |
| South | Basilicata | Potenza | 90+ | 329 | 280 (224 to 334) | 49 (-5 to 105) | 1.17 (0.98 to 1.47) |
| South | Calabria | Catanzaro | 0 to 59 | 69 | 64 (58 to 71) | 5 (-2 to 11) | 1.07 (0.97 to 1.19) |
| South | Calabria | Catanzaro | 60 to 69 | 104 | 93 (78 to 111) | 11 (-7 to 26) | 1.11 (0.94 to 1.34) |
| South | Calabria | Catanzaro | 70 to 79 | 172 | 179 (148 to 212) | -7 (-40 to 24) | 0.96 (0.81 to 1.16) |
| South | Calabria | Catanzaro | 80 to 89 | 368 | 352 (279 to 425) | 16 (-57 to 89) | 1.05 (0.87 to 1.32) |
| South | Calabria | Catanzaro | 90+ | 227 | 221 (175 to 265) | 6 (-38 to 52) | 1.03 (0.86 to 1.30) |
| South | Calabria | Cosenza | 0 to 59 | 147 | 145 (134 to 158) | 2 (-11 to 13) | 1.01 (0.93 to 1.10) |
| South | Calabria | Cosenza | 60 to 69 | 194 | 187 (157 to 221) | 7 (-27 to 37) | 1.04 (0.88 to 1.24) |
| South | Calabria | Cosenza | 70 to 79 | 386 | 350 (288 to 411) | 36 (-25 to 98) | 1.10 (0.94 to 1.34) |
| South | Calabria | Cosenza | 80 to 89 | 832 | 759 (598 to 921) | 73 (-89 to 234) | 1.10 (0.90 to 1.39) |
| South | Calabria | Cosenza | 90+ | 460 | 418 (335 to 499) | 42 (-39 to 125) | 1.10 (0.92 to 1.38) |
| South | Calabria | Crotone | 0 to 59 | 32 | 40 (36 to 46) | -8 (-14 to -4) | 0.79 (0.70 to 0.90) |

| Macroregion | Region | Province | Age group | Observed deaths | Expected baseline | Excess deaths | SMRs |
|---|---|---|---|---|---|---|---|
| South | Calabria | Crotone | 60 to 69 | 43 | 45 (37 to 55) | -2 (-12 to 6) | 0.95 (0.78 to 1.18) |
| South | Calabria | Crotone | 70 to 79 | 114 | 84 (69 to 101) | 30 (13 to 45) | 1.36 (1.13 to 1.66) |
| South | Calabria | Crotone | 80 to 89 | 178 | 167 (130 to 201) | 11 (-23 to 48) | 1.07 (0.89 to 1.37) |
| South | Calabria | Crotone | 90+ | 95 | 75 (59 to 90) | 20 (5 to 36) | 1.27 (1.05 to 1.61) |
| South | Calabria | Reggio di Calabria | 0 to 59 | 100 | 102 (93 to 111) | -2 (-11 to 7) | 0.99 (0.90 to 1.08) |
| South | Calabria | Reggio di Calabria | 60 to 69 | 160 | 131 (110 to 155) | 29 (5 to 50) | 1.22 (1.03 to 1.45) |
| South | Calabria | Reggio di Calabria | 70 to 79 | 300 | 268 (221 to 317) | 32 (-17 to 79) | 1.12 (0.95 to 1.35) |
| South | Calabria | Reggio di Calabria | 80 to 89 | 598 | 549 (432 to 659) | 49 (-61 to 166) | 1.09 (0.91 to 1.38) |
| South | Calabria | Reggio di Calabria | 90+ | 397 | 355 (284 to 430) | 42 (-33 to 113) | 1.12 (0.92 to 1.40) |
| South | Calabria | Vibo Valentia | 0 to 59 | 26 | 31 (27 to 36) | -5 (-10 to -1) | 0.83 (0.72 to 0.97) |
| South | Calabria | Vibo Valentia | 60 to 69 | 49 | 39 (31 to 48) | 10 (1 to 18) | 1.25 (1.03 to 1.56) |
| South | Calabria | Vibo Valentia | 70 to 79 | 69 | 81 (66 to 98) | -12 (-29 to 3) | 0.85 (0.71 to 1.04) |
| South | Calabria | Vibo Valentia | 80 to 89 | 160 | 155 (122 to 188) | 5 (-28 to 38) | 1.03 (0.85 to 1.31) |
| South | Calabria | Vibo Valentia | 90+ | 111 | 93 (74 to 114) | 18 (-3 to 37) | 1.19 (0.98 to 1.50) |
| South | Campania | Avellino | 0 to 59 | 109 | 82 (75 to 90) | 27 (19 to 34) | 1.32 (1.21 to 1.45) |
| South | Campania | Avellino | 60 to 69 | 147 | 108 (89 to 129) | 39 (18 to 58) | 1.37 (1.14 to 1.65) |
| South | Campania | Avellino | 70 to 79 | 217 | 218 (178 to 258) | -1 (-41 to 39) | 1.00 (0.84 to 1.22) |
| South | Campania | Avellino | 80 to 89 | 507 | 456 (360 to 545) | 51 (-38 to 147) | 1.11 (0.93 to 1.41) |
| South | Campania | Avellino | 90+ | 339 | 307 (245 to 367) | 32 (-28 to 94) | 1.10 (0.92 to 1.38) |
| South | Campania | Benevento | 0 to 59 | 54 | 56 (50 to 62) | -2 (-8 to 4) | 0.97 (0.88 to 1.07) |
| South | Campania | Benevento | 60 to 69 | 76 | 73 (61 to 88) | 3 (-12 to 15) | 1.03 (0.86 to 1.25) |
| South | Campania | Benevento | 70 to 79 | 138 | 145 (119 to 172) | -7 (-34 to 19) | 0.95 (0.80 to 1.16) |
| South | Campania | Benevento | 80 to 89 | 304 | 316 (249 to 381) | -12 (-77 to 55) | 0.96 (0.80 to 1.22) |
| South | Campania | Benevento | 90+ | 226 | 197 (157 to 237) | 29 (-11 to 69) | 1.15 (0.95 to 1.44) |
| South | Campania | Caserta | 0 to 59 | 186 | 215 (201 to 229) | -29 (-43 to -15) | 0.87 (0.81 to 0.92) |
| South | Campania | Caserta | 60 to 69 | 231 | 243 (202 to 285) | -12 (-54 to 29) | 0.95 (0.81 to 1.14) |
| South | Campania | Caserta | 70 to 79 | 493 | 461 (383 to 543) | 32 (-50 to 110) | 1.07 (0.91 to 1.29) |
| South | Campania | Caserta | 80 to 89 | 707 | 735 (581 to 887) | -28 (-180 to 126) | 0.96 (0.80 to 1.22) |
| South | Campania | Caserta | 90+ | 355 | 342 (272 to 408) | 13 (-53 to 83) | 1.04 (0.87 to 1.31) |
| South | Campania | Napoli | 0 to 59 | 747 | 704 (671 to 737) | 43 (10 to 76) | 1.06 (1.01 to 1.11) |
| South | Campania | Napoli | 60 to 69 | 874 | 846 (711 to 988) | 28 (-114 to 163) | 1.03 (0.88 to 1.23) |
| South | Campania | Napoli | 70 to 79 | 1,703 | 1,605 (1,333 to 1,887) | 98 (-184 to 370) | 1.06 (0.90 to 1.28) |
| South | Campania | Napoli | 80 to 89 | 2,367 | 2,457 (1,944 to 2,948) | -90 (-581 to 423) | 0.96 (0.80 to 1.22) |
| South | Campania | Napoli | 90+ | 1,179 | 1,120 (893 to 1,345) | 59 (-166 to 286) | 1.05 (0.88 to 1.32) |
| South | Campania | Salerno | 0 to 59 | 241 | 235 (219 to 250) | 6 (-9 to 22) | 1.03 (0.96 to 1.10) |
| South | Campania | Salerno | 60 to 69 | 280 | 289 (241 to 343) | -9 (-63 to 39) | 0.97 (0.82 to 1.16) |
| South | Campania | Salerno | 70 to 79 | 578 | 543 (447 to 643) | 35 (-65 to 131) | 1.06 (0.90 to 1.29) |
| South | Campania | Salerno | 80 to 89 | 1,060 | 1,104 (876 to 1,330) | -44 (-270 to 184) | 0.96 (0.80 to 1.21) |
| South | Campania | Salerno | 90+ | 608 | 643 (513 to 776) | -35 (-168 to 95) | 0.95 (0.78 to 1.19) |
| South | Molise | Campobasso | 0 to 59 | 30 | 37 (33 to 42) | -7 (-12 to -3) | 0.81 (0.72 to 0.91) |
| South | Molise | Campobasso | 60 to 69 | 68 | 58 (47 to 70) | 10 (-2 to 21) | 1.18 (0.97 to 1.43) |
| South | Molise | Campobasso | 70 to 79 | 121 | 122 (102 to 147) | -1 (-26 to 19) | 0.99 (0.83 to 1.19) |
| South | Molise | Campobasso | 80 to 89 | 293 | 263 (207 to 319) | 30 (-26 to 86) | 1.11 (0.92 to 1.41) |
| South | Molise | Campobasso | 90+ | 191 | 177 (140 to 214) | 14 (-23 to 51) | 1.08 (0.89 to 1.37) |

| Macroregion | Region | Province | Age group | Observed deaths | Expected baseline | Excess deaths | SMRs |
|---|---|---|---|---|---|---|---|
| South | Molise | Isernia | 0 to 59 | 15 | 15 (13 to 18) | 0 (-3 to 2) | 0.97 (0.82 to 1.16) |
| South | Molise | Isernia | 60 to 69 | 22 | 25 (20 to 31) | -3 (-9 to 2) | 0.89 (0.71 to 1.11) |
| South | Molise | Isernia | 70 to 79 | 46 | 47 (38 to 57) | -1 (-11 to 8) | 0.98 (0.80 to 1.21) |
| South | Molise | Isernia | 80 to 89 | 118 | 112 (88 to 136) | 6 (-18 to 30) | 1.06 (0.87 to 1.34) |
| South | Molise | Isernia | 90+ | 85 | 80 (63 to 98) | 5 (-13 to 22) | 1.06 (0.87 to 1.36) |
| South | Puglia | Bari | 0 to 59 | 224 | 210 (196 to 225) | 14 (-1 to 28) | 1.07 (0.99 to 1.14) |
| South | Puglia | Bari | 60 to 69 | 304 | 267 (224 to 316) | 37 (-12 to 80) | 1.14 (0.96 to 1.36) |
| South | Puglia | Bari | 70 to 79 | 639 | 555 (458 to 651) | 84 (-12 to 181) | 1.15 (0.98 to 1.39) |
| South | Puglia | Bari | 80 to 89 | 1,204 | 1,110 (880 to 1,338) | 94 (-134 to 324) | 1.08 (0.90 to 1.37) |
| South | Puglia | Bari | 90+ | 815 | 678 (542 to 813) | 137 (2 to 273) | 1.20 (1.00 to 1.50) |
| South | Puglia | Barletta-Andria-Trani | 0 to 59 | 79 | 75 (68 to 82) | 4 (-3 to 11) | 1.05 (0.96 to 1.16) |
| South | Puglia | Barletta-Andria-Trani | 60 to 69 | 83 | 83 (69 to 98) | 0 (-15 to 14) | 1.00 (0.85 to 1.20) |
| South | Puglia | Barletta-Andria-Trani | 70 to 79 | 197 | 177 (145 to 208) | 20 (-11 to 52) | 1.11 (0.95 to 1.35) |
| South | Puglia | Barletta-Andria-Trani | 80 to 89 | 378 | 330 (260 to 396) | 48 (-18 to 118) | 1.15 (0.95 to 1.45) |
| South | Puglia | Barletta-Andria-Trani | 90+ | 211 | 168 (134 to 202) | 43 (9 to 77) | 1.26 (1.05 to 1.58) |
| South | Puglia | Brindisi | 0 to 59 | 75 | 72 (65 to 80) | 3 (-5 to 10) | 1.04 (0.94 to 1.15) |
| South | Puglia | Brindisi | 60 to 69 | 96 | 92 (77 to 110) | 4 (-14 to 19) | 1.04 (0.87 to 1.25) |
| South | Puglia | Brindisi | 70 to 79 | 251 | 206 (170 to 244) | 45 (7 to 81) | 1.22 (1.03 to 1.48) |
| South | Puglia | Brindisi | 80 to 89 | 481 | 407 (319 to 494) | 74 (-13 to 162) | 1.18 (0.97 to 1.51) |
| South | Puglia | Brindisi | 90+ | 276 | 229 (180 to 277) | 47 (-1 to 96) | 1.20 (1.00 to 1.53) |
| South | Puglia | Foggia | 0 to 59 | 134 | 126 (116 to 135) | 8 (-1 to 18) | 1.07 (0.99 to 1.15) |
| South | Puglia | Foggia | 60 to 69 | 159 | 143 (119 to 168) | 16 (-9 to 40) | 1.11 (0.95 to 1.33) |
| South | Puglia | Foggia | 70 to 79 | 341 | 295 (245 to 349) | 46 (-8 to 96) | 1.15 (0.98 to 1.39) |
| South | Puglia | Foggia | 80 to 89 | 717 | 592 (470 to 709) | 125 (8 to 247) | 1.21 (1.01 to 1.52) |
| South | Puglia | Foggia | 90+ | 452 | 360 (287 to 430) | 92 (22 to 165) | 1.25 (1.05 to 1.58) |
| South | Puglia | Lecce | 0 to 59 | 166 | 132 (122 to 143) | 34 (23 to 44) | 1.26 (1.16 to 1.37) |
| South | Puglia | Lecce | 60 to 69 | 189 | 187 (157 to 222) | 2 (-33 to 32) | 1.01 (0.85 to 1.21) |
| South | Puglia | Lecce | 70 to 79 | 453 | 418 (346 to 490) | 35 (-37 to 107) | 1.08 (0.92 to 1.31) |
| South | Puglia | Lecce | 80 to 89 | 904 | 851 (672 to 1,023) | 53 (-119 to 232) | 1.06 (0.88 to 1.35) |
| South | Puglia | Lecce | 90+ | 563 | 520 (413 to 624) | 43 (-61 to 150) | 1.08 (0.90 to 1.36) |
| South | Puglia | Taranto | 0 to 59 | 102 | 100 (91 to 109) | 2 (-7 to 11) | 1.02 (0.93 to 1.12) |
| South | Puglia | Taranto | 60 to 69 | 132 | 130 (109 to 154) | 2 (-22 to 23) | 1.02 (0.86 to 1.21) |
| South | Puglia | Taranto | 70 to 79 | 317 | 295 (241 to 348) | 22 (-31 to 76) | 1.08 (0.91 to 1.31) |
| South | Puglia | Taranto | 80 to 89 | 584 | 560 (445 to 674) | 24 (-90 to 139) | 1.04 (0.87 to 1.31) |
| South | Puglia | Taranto | 90+ | 364 | 314 (251 to 375) | 50 (-11 to 113) | 1.16 (0.97 to 1.45) |

## References


1. Italian National Institute of Statistics. IStat database. https://dati.istat.it (2020).

2. Danielson, J. J. & Gesch, D. B. *Global Multi-resolution Terrain Elevation Data 2010 (GMTED2010): U.S. Geological Survey Open-File Report 2011-1073*. https://pubs.usgs.gov/of/2011/1073/ (2011).

3. Weiss, D. J. *et al.* Global maps of travel time to healthcare facilities. *Nat. Med.* **26**, 1835–1838 (2020).

4. Lamprecht, C. Meteostat API. *Meteostat* https://meteostat.net (2021).

5. Italian Meteorological Service. Web Portal: Military Air Force Meteorological Service. http://meteoam.it/ (2020).

6. Wang, H. *et al.* Global, regional, and national life expectancy, all-cause mortality, and cause-specific mortality for 249 causes of death, 1980–2015: a systematic analysis for the Global Burden of Disease Study 2015. *Lancet* **388**, 1459–1544 (2016).

7. Roelfs, D. J., Shor, E., Davidson, K. W. & Schwartz, J. E. Losing life and livelihood: A systematic review and meta-analysis of unemployment and all-cause mortality. *Soc. Sci. Med.* **72**, 840–854 (2011).

8. Stuckler, D., Basu, S. & McKee, M. Budget crises, health, and social welfare programmes. *BMJ* **340**, c3311–c3311 (2010).

9. Bergqvist, K., Yngwe, M. Å. & Lundberg, O. Understanding the role of welfare state characteristics for health and inequalities - An analytical review. *BMC Public Health* **13**, (2013).

10. Osler, M. *et al.* Income inequality, individual income, and mortality in Danish adults: analysis of pooled data from two cohort studies. *BMJ* **324**, 13–13 (2002).

11. Nicholl, J., West, J., Goodacre, S. & Turner, J. The relationship between distance to hospital and patient mortality in emergencies: An observational study. *Emerg. Med. J.* **24**, 665–668 (2007).

12. Burtscher, M. Effects of living at higher altitudes on mortality: A narrative review. *Aging Dis.* **5**, 274–280 (2014).

13. Yu, W. *et al.* Daily average temperature and mortality among the elderly: A meta-analysis and systematic review of epidemiological evidence. *Int. J. Biometeorol.* **56**, 569–581 (2012).
